# Subject Index Volume 13

**DOI:** 10.1111/jdi.13956

**Published:** 2022-12-04

2A protease

Immunohistochemical detection of enteroviruses in pancreatic tissues of patients with type 1 diabetes using a polyclonal antibody against 2A protease of Coxsackievirus, Jimbo 435–442.

2p25.3 deletion

A Japanese patient with a 2p25.3 terminal deletion presented with early‐onset obesity, intellectual disability and diabetes mellitus: A case report, Sakaue 391–396.

50‐Gram glucose challenge test

Association of leukocyte counts in the first trimester with glucose intolerance during pregnancy, Wang 191–200.

α‐Thalassemia

Discordantly high glycated hemoglobin might assist in diagnosing α‐thalassemia, but not diabetes: A case report, Gao 1633–1635.

β‐Cell

β‐Cell replacement therapy for the cure of diabetes, Lee 1798–1802.

β‐Cell function

Time in range assessed by capillary blood glucose in relation to insulin sensitivity and β‐cell function in patients with type 2 diabetes mellitus: A cross‐sectional study in China, Ye 1825–1833.

β‐Cell mass maintenance

Roles of FoxM1‐driven basal β‐cell proliferation in maintenance of β‐cell mass and glucose tolerance during adulthood, Kohata 1666–1676.

β‐Cell proliferation

Roles of FoxM1‐driven basal β‐cell proliferation in maintenance of β‐cell mass and glucose tolerance during adulthood, Kohata 1666–1676.

*ABCG5*

Plant sterol hyperabsorption caused by uncontrolled diabetes in a patient with a heterozygous *ABCG5* variant, Hasebe 1934–1938.

Abdominal aortic calcification

Abdominal aortic calcification is associated with Fibrosis‐4 index and low body mass index in type 2 diabetes patients: A retrospective cross‐sectional study, Togashi 1861–1872.

Abdominal fat

Abdominal volume index trajectories and risk of diabetes mellitus: Results from the China Health and Nutrition Survey, Hu 868–877.

Acceptance and commitment therapy

Efficacy of acceptance and commitment therapy for people with type 2 diabetes: Systematic review and meta‐analysis, Sakamoto 262–270.

Acute kidney injury

Acute kidney injury in Japanese type 2 diabetes patients receiving sodium–glucose cotransporter 2 inhibitors: A nationwide cohort study, Horii 42–46.

Adaptive immune system

Role of the adaptive immune system in diabetic kidney disease, Kong 213–226.

Adenosine A1 receptor inhibitor

Adenosine/adenosine type 1 receptor signaling pathway did not play dominant roles on the influence of sodium–glucose cotransporter 2 inhibitor in the kidney of bovine serum albumin‐overloaded streptozotocin‐induced diabetic mice, Shimada 955–964.

Adherence to healthy lifestyles overtime

Improving and maintaining healthy lifestyles are associated with a lower risk of diabetes: A large cohort study, Kuwahara 714–724.

Adipocytokines,

The relationship between serum adipokine fibroblast growth factor‐21 and gestational diabetes mellitus, Jia 2047–2053.

Adipokine

Circulating level of fatty acid‐binding protein 4is an independent predictor of metabolic dysfunction‐associated fatty liver disease in middle‐aged and elderly individuals, Tanaka 878–888.

Adiponectin

Hyperlipidemic plasma molecules bind and inhibit adiponectin activity, Zhang 947–954.
Severe aortic stenosis during leptin replacement therapy in a patient with generalized lipodystrophy‐associated progeroid syndrome due to an LMNA variant: A case report, Sasako 1636–1638.

Adiposity rebound

Investigation of umbilical cord serum miRNAs associated with childhood obesity: A pilot study from a birth cohort study, Takatani 1740–1744.

Adjuvant chemotherapy

Association between diabetes and adjuvant chemotherapy implementation in patients with stage III colorectal cancer, Kanehara 1771–1778.

Administrative claims data

Appropriate definition of diabetes using an administrative database: A cross‐sectional cohort validation study, Nishioka 249–255.
The age of death in Japanese patients with type 2 and type 1 diabetes: A descriptive epidemiological study, Nishioka 1316–1320.

Administrative claims database

Association between dipeptidyl peptidase‐4 inhibitors and increased risk for bullous pemphigoid within 3 months from first use: A 5‐year population‐based cohort study using the Japanese National Database, Kuwata 460–467.

Adolescent

Clinical course of adolescents with type 2 diabetes mellitus: A nationwide cohort study in Taiwan, Yen 1905–1913.

Advancing age

Roles of FoxM1‐driven basal β‐cell proliferation in maintenance of β‐cell mass and glucose tolerance during adulthood, Kohata 1666–1676.

Advocacy

A survey of clinical physician's perceptions of stigma and advocacy in patients with type 2 diabetes in Kanagawa, Japan, Matsuzawa 2073–2080.
Stigma evaluation for diabetes and other chronic non‐communicable disease patients: Development, validation and clinical use of stigma scale–The Kanden Institute Stigma Scale, Tanaka 2081–2090.

Age of death

The age of death in Japanese patients with type 2 and type 1 diabetes: A descriptive epidemiological study, Nishioka 1316–1320.

Aging

Treatment of diabetic macular edema in real‐world clinical practice: The effect of aging, Kusuhara 1339–1346.

Albuminuria

Visit‐to‐visit variability in albuminuria predicts renal function deterioration in patients with type 2 diabetes, Lin 1021–1029.

American Heart Association

Association of ideal cardiovascular health metrics and incident type 2 diabetes mellitus among an urban population of Iran: One decade follow up in the Tehran Lipid and Glucose Study, Asgari 1711–1722.

Amputation

When amputation is not the end of the challenge: A successful therapy for osteomyelitis and soft tissue infection in a patient with type 1 diabetes, Caruso 209–212.

Amyloid

Diversity of pathophysiology in type 2 diabetes shown by islet pathology, Mizukami 6–13.

Amyloidosis

Repeated insulin injection without site rotation affects skin thickness – ultrasonographic and histological evaluation, Murao 997–1003.

Anemia

Sodium–glucose cotransporter 2 inhibitors and anemia among diabetes patients in real clinical practice, Murashima 638–646.

Ankle‐brachial index

The early diagnostic value of ankle‐brachial index combined with feet electrochemical skin conductance for peripheral artery disease in type 2 diabetes, Zhao 525–531.

Antidiabetic agents

Retrospective nationwide study on the trends in first‐line antidiabetic medication for patients with type 2 diabetes in Japan, Bouchi 280–291.

Antidiabetic drugs

Current status of oral antidiabetic drug prescribing patterns based on the body mass index for Japanese type 2 diabetes mellitus patients and yearly changes in diabetologists' prescribing patterns from 2002 to 2019 (JDDM61), Yagi 65–73.

Anti‐insulin antibody

Serious diabetic ketoacidosis induced by insulin allergy and anti‐insulin antibody in an individual with type 2 diabetes mellitus, Takahashi 1788–1792.

Area under the curve in range

Comparison of glucose time in range and area under curve in range in relation to risk of diabetic retinopathy in type 2 diabetes patients, Wang 1543–1550.

Artificial pancreas systems

Use of automated insulin delivery systems in people with type 1 diabetes fasting during Ramadan: An observational study, Aldibbiat 647–651.

Asia

Healthcare resource utilization in patients treated with empagliflozin in East Asia, Sheu 810–821.

ATP2B1‐AS1

Long non‐coding ribonucleic acid ATP2B1‐AS1modulates endothelial permeability through regulating the miR‐4,729–IQGAP2 axis in diabetic retinopathy, Ren 443–452.

Autoimmune pancreatitis

Factors affecting glycemic control in diabetes mellitus complicated by autoimmune pancreatitis, Harai 1387–1395.

Bariatric surgery

Bariatric surgery versus medical treatment in mildly obese patients with type 2 diabetes mellitus in Japan: Propensity score‐matched analysis on real‐world data, Seki 74–84.
East Asian perspectives in metabolic and bariatric surgery, Oh 756–761.

Basal insulin

Basal insulin requirement in patients with type1 diabetes depends on the age and body mass index, Mitsui 292–298.

Blood pressure

Impact of the first announced state of emergency owing to coronavirus disease 2019 on stress and blood pressure levels among patients with type 2 diabetes mellitus in Japan, Ito 1607–1616.

Body composition

Comparison of the effects of frequent versus conventional nutritional interventions inpatients with type 2 diabetes mellitus: A randomized, controlled trial, Kawabata 271–279.
Association between fat mass index, fat‐free mass index and hemoglobin A1c in a Japanese population: The Tohoku Medical Megabank Community‐based Cohort Study, Takase 858–867.

Body composition

Year‐long effects of COVID‐19 restrictions on glycemic control and body composition inpatients with glucose intolerance in Japan: A single‐center retrospective study, Tsukaguchi 2063–2072.

Body mass index

Current status of oral antidiabetic drug prescribing patterns based on the body mass index for Japanese type 2 diabetes mellitus patients and yearly changes in diabetologists' prescribing patterns from 2002 to 2019 (JDDM61), Yagi 65–73.
The association between weight gain at different stages of pregnancy and risk of gestational diabetes mellitus, Chuang 359–366.
Association between pre‐pregnancy body mass index and gestational weight gain and perinatal outcomes in pregnant women diagnosed with gestational diabetes mellitus: The Japan Environment and Children's Study, Saito 889–899.
Efficacy and safety of once‐weekly semaglutide in Japanese individuals with type 2 diabetes by baseline age and body mass index, Yabe 1161–1174.
Association of body mass index with risk of prediabetes in Chinese adults: A population‐based cohort study, Chai 1235–1244.

Bodyweight reduction

Association between changes in bodyweight and cardiovascular disease risk factors among obese Japanese patients with type 2 diabetes, Yoshimura 1560–1566.

Bone mineral density

Association between muscle mass, bone mineral density and osteoporosis in type 2 diabetes, Pan 351–358.

Bone mineral density

Relation between the insulin lowering rate and changes in bone mineral density: Analysis among subtypes of type 1 diabetes mellitus, Suzuki 1585–1595.

Bullous pemphigoid

Association between dipeptidyl peptidase‐4 inhibitors and increased risk for bullous pemphigoid within 3 months from first use: A 5‐year population‐based cohort study using the Japanese National Database, Kuwata 460–467.

C1q tumor necrosis factor‐related protein 4

C1q tumor necrosis factor‐related protein 4 is associated with coronary artery disease in patients with type 2 diabetes, Gao 1723–1731.

Canagliflozin

Renal, cardiovascular and safety outcomes of canagliflozin in patients with type 2 diabetes and nephropathy in East and South‐East Asian countries: Results from the Canagliflozin and Renal Events in Diabetes with Established Nephropathy Clinical Evaluation Trial, Wada 54–64.
Effect of canagliflozin on the decline of estimated glomerular filtration rate in chronic kidney disease patients with type 2 diabetes mellitus: A multicenter, randomized, double‐blind, placebo‐controlled, parallel‐group, phase III study in Japan, Wada 1981–1989.

Cardiac dysfunction

Association between cortisol and left ventricular diastolic dysfunction in patients with diabetes mellitus, Sagara 344–350.

Cardiometabolic index

Transition of cardiometabolic status and the risk of type 2 diabetes mellitus among middle‐aged and older Chinese: A national cohort study, Qiu 1426–1437.

Cardiovascular autonomic neuropathy

Decline in renal function associated with cardiovascular autonomic neuropathy positively coordinated with proteinuria in patients with type 2 diabetes, Muramatsu 102–111.
Utility of using electrocardiogram measures of heart rate variability as a measure of cardiovascular autonomic neuropathy in type 1 diabetes patients, Pop‐Busui 125–133.
Point‐of‐care sural nerve conduction could predict the presence of cardiovascular autonomic neuropathy in type 1 diabetes mellitus, Nattero‐Chávez 1347–1356.

Cardiovascular disease

Cardiovascular events and atherosclerosis inpatients with type 2 diabetes and impaired glucose tolerance: What are the medical treatments to prevent cardiovascular events in such patients?, Ishii 1114–1121.
Treatment of diabetes mellitus has borne much fruit in the prevention of cardiovascular disease, Yagyu 1472–1488.

Cardiovascular reflex tests,

Utility of using electrocardiogram measures of heart rate variability as a measure of cardiovascular autonomic neuropathy in type 1diabetes patients, Pop‐Busui 125–133.

Cardiovascular disease risk factors

Association between changes in bodyweight and cardiovascular disease risk factors among obese Japanese patients with type 2 diabetes, Yoshimura 1560–1566.

Case detection

Non‐laboratory‐based risk assessment model for case detection of diabetes mellitus and pre‐diabetes in primary care, Dong 1374–1386.

Caspase‐1

VX‐765 ameliorates renal injury and fibrosis in diabetes by regulating caspase‐1‐mediated pyroptosis and inflammation, Wen 22–33.

CCM

Corneal confocal microscopy for the diagnosis of diabetic peripheral neuropathy: A systematic review and meta‐analysis, Gad 134–147.

Childhood obesity

Investigation of umbilical cord serum miRNAs associated with childhood obesity: A pilot study from a birth cohort study, Takatani 1740–1744.

Chinese

Association of body mass index with risk of prediabetes in Chinese adults: A population‐based cohort study, Chai 1235–1244.

Cholesterol efflux capacity

Cholesterol efflux capacity of high‐density lipoprotein was not associated with cognitive decline and brain structures in older people with diabetes mellitus, Wu 1873–1880.

Chronic disease

Stigma evaluation for diabetes and other chronic non‐communicable disease patients: Development, validation and clinical use of stigma scale–The Kanden Institute Stigma Scale, Tanaka 2081–2090.

Chronic heart failure

Effect of canagliflozin on white blood cell counts in patients with type 2 diabetes and heart failure: A subanalysis of the randomized CANDLE trial, Tanaka 1990–1999.

Chronic kidney disease

Dipeptidyl peptidase‐4 inhibitor and insulin combination treatment in type 2 diabetes and chronic kidney disease: A meta‐analysis, Zhou 468–477.
The renoprotective effect of once‐weekly GLP‐1 receptor agonist dulaglutide on progression of nephropathy in Japanese patients with type 2 diabetes and moderate to severe chronic kidney disease (JDDM67), Tsuchida 1834–1841.
Effect of canagliflozin on the decline of estimated glomerular filtration rate in chronic kidney disease patients with type 2 diabetes mellitus: A multicenter, randomized, double‐blind, placebo‐controlled, parallel‐group, phase III study in Japan, Wada 1981–1989.

Circular ribonucleic acid nucleoporin 98

Circular ribonucleic acid nucleoporin 98 knockdown alleviates high glucose‐induced proliferation, fibrosis, inflammation and oxidative stress in human glomerular mesangial cells by regulating the microribonucleic acid‐151‐3p–high mobility group AT‐hook 2 axis, Dong 1303–1315.

Claims database

Effect of sodium–glucose cotransporter 2 inhibitor medication on new prescriptions of antihypertensives, antigout/antihyperuricemics and antidyslipidemics in Japan: Analysis using the JMDC Claims Database, Gouda 1842–1851.

Classified stigmas

A survey of clinical physician's perceptions of stigma and advocacy in patients with type 2 diabetes in Kanagawa, Japan, Matsuzawa 2073–2080.

Coefficient of variation of the R‐R interval

Decline in renal function associated with cardiovascular autonomic neuropathy positively coordinated with proteinuria in patients with type 2 diabetes, Muramatsu 102–111.

Coffee

Relationship of coffee consumption with a decline in kidney function among patients with type 2 diabetes: The Fukuoka Diabetes Registry, Komorita 1030–1038.

Cognitive dysfunction

Voxel‐based specific regional analysis system for Alzheimer's disease utility as a screening tool for unrecognized cognitive dysfunction of elderly patients in diabetes outpatient clinics: Multicenter retrospective exploratory study, Ueba 177–184.

Cohort studies

Association of glycated hemoglobin at an early stage of pregnancy with the risk of gestational diabetes mellitus among non‐diabetic women in Japan: The Japan Environment and Children's Study, Sekine 687–695.

Cohort study

Improving and maintaining healthy lifestyles are associated with a lower risk of diabetes: A large cohort study, Kuwahara 714–724.
Transition of cardiometabolic status and the risk of type 2 diabetes mellitus among middle‐aged and older Chinese: A national cohort study, Qiu 1426–1437.
Association between maternal gestational diabetes mellitus and high‐sensitivity C‐reactive protein levels in 8‐year‐old children: The Yamanashi Adjunct Study of the Japan Environment and Children's Study(JECS), Sekine 1444–1447.

Colorectal cancer

Association between diabetes and adjuvant chemotherapy implementation in patients with stage III colorectal cancer, Kanehara 1771–1778.

Commentary

Astrocyte glucose metabolism highlights the link between cannabis use and social behavior, Chanda 14–16.
Regulation of food intake by intestinal hormones in brain, Harada 17–18.
Metformin induces insulin secretion by preserving pancreatic aquaporin 7 expression in type 2 diabetes mellitus, Kimura 227–229.
Diabetes duration and obesity matter in autologous mesenchymal stem/stromal cell transplantation in type 2 diabetes patients, Lee 230–232.
Unmet needs in current clinical practice for insulinoma: Lessons from nationwide studies in Japan, Inagaki 429–431.
Trained immunity: A key player of “metabolic memory” in diabetes, Naruse 608–610.
Research following genome‐wide association study focuses on the multifaceted nature of Src kinase‐associated phosphoprotein 2 in type 1diabetes, Sano 611–613.
Assessing liver fibrosis in all patients with type 2 diabetes and fatty liver disease: It's time to act now, Lam 762–764.
Similarities and differences in the natural history of youth‐onset type 2 diabetes between the West and Asia, Hwu 941–943.
CREBH regulation of lipid metabolism through multifaceted functions that improve arteriosclerosis, Nakagawa 1129–1131.
Pick the best of both glucose and lipid metabolism, Shimizu 1132–1133.
Clinical relevance of dual agonist of glucagon and glucagon‐like peptide‐1 receptors to achieve functional restoration of first‐ and second‐phase insulin secretion, Kimura 1300–1302.
Identification of islet cell characteristics in humans with type 2 diabetes by single‐cell sequencing, Imai 1646–1648.
Hyperglycemia‐mediated oocyte TET3 insufficiency predisposes offspring to glucose intolerance, Wu 1649–1651.
Improvement of hepatic steatosis and fibrosis in diabetes: Which bariatric procedure is more appropriate?, Jia 1803–1804.
Potential of a glucagon‐like peptide‐1 receptor/glucose‐dependent insulinotropic polypeptide receptor/glucagon receptor triagonist for the treatment of obesity and type 2 diabetes, Araki 1958–1960.

Commuting

Active commuting, commuting modes and the risk of diabetes: 14‐year follow‐up data from the Hisayama study, Honda 1677–1684.

Complement system

Activation of complement C1q and C3 in glomeruli might accelerate the progression of diabetic nephropathy: Evidence from transcriptomic data and renal histopathology, Jiao 839–849.

Concomitant medication

Effect of sodium–glucose cotransporter 2 inhibitor medication on new prescriptions of antihypertensives, antigout/antihyperuricemics and antidyslipidemics in Japan: Analysis using the JMDC Claims Database, Gouda 1842–1851.

Congenital heart defect

Pregestational diabetes mediates the association between maternal obesity and the risk of congenital heart defects, Wu 367–374.

Connectivity map analysis

Application of weighted gene co‐expression network analysis to identify novel key genes in diabetic nephropathy, Wang 112–124.

Constipation

Effects of probiotic *Bifidobacterium bifidum* G9‐1 on the gastrointestinal symptoms of patients with type 2 diabetes mellitus treated with metformin: An open‐label, single‐arm, exploratory research trial, Hata 489–500.

Continuous glucose monitoring

Association of glycemic variability assessed by continuous glucose monitoring with subclinical diabetic polyneuropathy in type 2 diabetes patients, Pan 328–335.
Comparison of glucose time in range and area under curve in range in relation to risk of diabetic retinopathy in type 2 diabetes patients, Wang 1543–1550.
Comparison of the clinical effects of intermittently scanned and real‐time continuous glucose monitoring in children and adolescents with type 1 diabetes: A retrospective cohort study, Urakami 1745–1752.
Continuous glucose monitoring system profile of women diagnosed as gestational diabetes mellitus by International Association of Diabetes and Pregnancy Study Groups criteria and labeled as normoglycemic by alternate criteria in early pregnancy, Gupta 1753–1760.

Continuous subcutaneous insulin infusion

Comparison of continuous subcutaneous insulin infusion treatment and multiple daily injection treatment on the progression of diabetic complications in Japanese patients with juvenile‐onset type 1 diabetes mellitus, Masuda 1528–1532.

Controlled study

Treatment of diabetes mellitus has borne much fruit in the prevention of cardiovascular disease, Yagyu 1472–1488.

Copper/zinc ratio

Synergistic association of the copper/zinc ratio under inflammatory conditions with diabetic kidney disease in patients with type 2 diabetes: The Asahi Diabetes Complications Study, Takao 299–307.

Coronary artery disease

C1q tumor necrosis factor‐related protein 4 is associated with coronary artery disease in patients with type 2 diabetes, Gao 1723–1731.

Corneal confocal microscopy

Progressive loss of corneal nerve fibers is associated with physical inactivity and glucose lowering medication associated with weight gain in type 2 diabetes, Ponirakis 1703–1710.

Coronavirus disease 2019

Lifestyle changes and their impact on glycemic control and weight control in patients with diabetes during the coronavirus disease 2019 pandemic in Japan, Takahara 375–385.
HbA1c level may be a risk factor for oxygen therapy requirement in patients with coronavirus disease 2019, Yanagisawa 909–917.
Impact of the first announced state of emergency owing to coronavirus disease 2019 on stress and blood pressure levels among patients with type 2 diabetes mellitus in Japan, Ito 1607–1616.

Cortisol

Association between cortisol and left ventricular diastolic dysfunction in patients with diabetes mellitus, Sagara 344–350.

COVID‐19

Impact of newly diagnosed diabetes on coronavirus disease 2019 severity and hyperglycemia, Uchihara 1086–1093.
Living and working environments are important determinants of glycemic control in patients with diabetes during the COVID‐19 pandemic: A retrospective observational study, Terakawa, 1094–1104.
Newly developed type 1 diabetes after coronavirus disease 2019 vaccination: A case report, Sasaki 1105–1108.
Impact of diabetes and Krebs von den Lungen‐6 on coronavirus disease 2019 severity: A single‐center study from Japan, Yakushiji 1277–1285.
Implementation of diabetes care and educational program via telemedicine inpatients with COVID‐19 in home isolation in Thailand: A real‐world experience, Harindhanavudhi 1448–1457.
Glycated hemoglobin level on admission associated with progression to severe disease in hospitalized patients with non‐severe coronavirus disease 2019, Numaguchi 1779–1787.
HbA1c measurement may save COVID‐19 inpatients from overlooked diabetes, Yoroidaka 1925–1933.
Year‐long effects of COVID‐19 restrictions on glycemic control and body composition inpatients with glucose intolerance in Japan: A single‐center retrospective study, Tsukaguchi 2063–2072.

COVID‐19 pandemic

Diabetes management by either telemedicine or clinic visit improved glycemic control during the coronavirus disease 2019 pandemic state of emergency in Japan, Onishi 386–390.
Substitution of telemedicine for clinic visit during the COVID‐19 pandemic of 2020: Comparison of telemedicine and clinic visit, Onishi 1617–1625.

COVID‐19 RNA‐based vaccines

Type 1 diabetes mellitus following COVID‐19 RNA‐based vaccine, Sakurai 1290–1292.

Cox proportional‐hazards regression model

Elevated gamma‐glutamyl transferase to high‐density lipoprotein cholesterol ratio has a non‐linear association with incident diabetes mellitus: A second analysis of a cohort study, Hu 2027–2037.

C‐peptide

Impact of flash glucose monitoring on glycemic control varies with the age and residual β‐cell function of patients with type 1 diabetes mellitus, Zhang 552–559.
Perioperative C‐peptide index is associated with the status of diabetes management after pancreatectomy, Shikata 1685–1694.

C‐peptide immunoreactivity

Comparisons of efficacy and safety in insulin glargine and lixisenatide plus glulisine combination therapy with multiple daily injection therapy in Japanese patients with type 2 diabetes, Kawaguchi 505–514.

CREDENCE

Renal, cardiovascular and safety outcomes of canagliflozin in patients with type 2 diabetes and nephropathy in East and South‐East Asian countries: Results from the Canagliflozin and Renal Events in Diabetes with Established Nephropathy Clinical Evaluation Trial, Wada 54–64.

Cyclic ADP ribose

A novel mechanism of imeglimin‐mediated insulin secretion via the cADPR‐TRP channel pathway, Funazaki 34–41.

Cyclin‐dependent kinase 5

Ocular expression of cyclin‐dependent kinase5 in patients with proliferative diabetic retinopathy, Sano 628–637.

Cytokines

Plasma cytokines during pregnancy provide insight into the risk of diabetes in the gestational diabetes risk group, Tagoma 1596–1606.

Database research

Fasting plasma glucose level in the range of 90–99 mg/dL and the risk of the onset of type 2 diabetes: Population‐based Panasonic cohort study 2, Munekawa 453–459.

Decline in the renal function

Decline in renal function associated with cardiovascular autonomic neuropathy positively coordinated with proteinuria in patients with type 2 diabetes, Muramatsu 102–111.

Dehydroepiandrosterone

Correlation of dehydroepiandrosterone with diabetic nephropathy and its clinical value in early detection, Ma 1695–1702.

Delivery of healthcare

Reimagining care for young adults living with type 1 diabetes, Morrissey 1294–1299.

Dementia

Cholesterol efflux capacity of high‐density lipoprotein was not associated with cognitive decline and brain structures in older people with diabetes mellitus, Wu 1873–1880.

Depression

Effect of depression on progression to end‐stage renal disease or pre‐end‐stage renal disease death in advanced diabetic nephropathy: A prospective cohort study of the Diabetes Study from the Center of Tokyo Women's Medical University, Horiba 94–101.
High prevalence of depressive symptoms among people with pediatric‐onset and adolescent‐onset type 1 diabetes: A cross‐sectional analysis of the Diabetes Study from the Center of Tokyo Women's Medical University, Takaike 1626–1632.

Diabetes

Lower handgrip strength levels probably precede triglyceride glucose index and associated with diabetes in men not in women, Zheng 148–155.
Effects of intermittent very‐low calorie diet on glycemic control and cardiovascular risk factors in obese patients with type 2 diabetes mellitus: A randomized controlled trial, Umphonsathien 156–166.
Mitochondrial haplogroups have a better correlation to insulin requirement than nuclear genetic variants for type 2 diabetes mellitus in Taiwanese individuals, Shen 201–208.
Appropriate definition of diabetes using an administrative database: A cross‐sectional cohort validation study, Nishioka 249–255.
Metabolic associated fatty liver disease is a risk factor for chronic kidney disease, Hashimoto 308–316.
Association between cortisol and left ventricular diastolic dysfunction in patients with diabetes mellitus, Sagara 344–350.
Impact of vision and hearing impairments on risk of cardiovascular outcomes and mortality in patients with type 2 diabetes: A nationwide cohort study, Jung 515–524.
Relationship between hemoglobin A1c level and flow‐mediated vasodilation in patients with type 2 diabetes mellitus receiving antidiabetic drugs, Yamaji 677–686.
Heart rate‐corrected QT interval: A novel diagnostic biomarker for diabetic peripheral neuropathy, Jiang 850–857.
Impact of newly diagnosed diabetes on coronavirus disease 2019 severity and hyperglycemia, Uchihara 1086–1093.
Cardiovascular events and atherosclerosis inpatients with type 2 diabetes and impaired glucose tolerance: What are the medical treatments to prevent cardiovascular events in such patients?, Ishii 1114–1121.
Impact of diabetes on coronary physiology evaluated by quantitative flow ratio in patients who underwent percutaneous coronary intervention, Ye 1203–1212.
Treatment of diabetic macular edema in real‐world clinical practice: The effect of aging, Kusuhara 1339–1346.
Obstructive sleep apnea, prediabetes and progression of type 2 diabetes: A systematic review and meta‐analysis, Wang 1396–1411.
Implementation of diabetes care and educational program via telemedicine inpatients with COVID‐19 in home isolation in Thailand: A real‐world experience, Harindhanavudhi 1448–1457.
Substitution of telemedicine for clinic visit during the COVID‐19 pandemic of 2020: Comparison of telemedicine and clinic visit, Onishi 1617–1625.
Discordantly high glycated hemoglobin might assist in diagnosing α‐thalassemia, but not diabetes: A case report, Gao 1633–1635.
Association between diabetes and adjuvant chemotherapy implementation in patients with stage III colorectal cancer, Kanehara 1771–1778.
Elevated gamma‐glutamyl transferase to high‐density lipoprotein cholesterol ratio has a non‐linear association with incident diabetes mellitus: A second analysis of a cohort study, Hu 2027–2037.

Diabetes education

Validity and reliability of the Japanese version of the diabetes knowledge test among in‐patients with type 2 diabetes, Minami 580–587.

Diabetes knowledge test

Validity and reliability of the Japanese version of the diabetes knowledge test among in‐patients with type 2 diabetes, Minami 580–587.

Diabetes mellitus

A Japanese patient with a 2p25.3 terminal deletion presented with early‐onset obesity, intellectual disability and diabetes mellitus: A case report, Sakaue 391–396.
Placental expression of glucose transporters GLUT‐1, GLUT‐3, GLUT‐8 and GLUT‐12 in pregnancies complicated by gestational and type 1 diabetes mellitus, Stanirowski 560–570.
Werner syndrome presenting as early‐onset diabetes: A case report, Wang 592–598.
Sodium–glucose cotransporter 2 inhibitors and anemia among diabetes patients in real clinical practice, Murashima 638–646.
Diabetes mellitus and its impact on mortality rate and outcome in pulmonary embolism, Volker H Schmitt 725–737.
Quantitative evaluation of insulin‐induced abdominal subcutaneous dystrophic tissue using shear wave elastography, Sato 1004–1010.
Abdominal volume index trajectories and risk of diabetes mellitus: Results from the China Health and Nutrition Survey, Hu 868–877.
Fatty liver with metabolic disorder, such as metabolic dysfunction‐associated fatty liver disease, indicates high risk for developing diabetes mellitus, Miyake 1245–1252.
Impact of diabetes and Krebs von den Lungen‐6 on coronavirus disease 2019 severity: A single‐center study from Japan, Yakushiji 1277–1285.
The age of death in Japanese patients with type 2 and type 1 diabetes: A descriptive epidemiological study, Nishioka 1316–1320.
Factors affecting glycemic control in diabetes mellitus complicated by autoimmune pancreatitis, Harai 1387–1395.
Effects of multi‐metal exposure on the risk of diabetes mellitus among people aged 40–75 years in rural areas in southwest China, Zhang 1412–1425.
Treatment of diabetes mellitus has borne much fruit in the prevention of cardiovascular disease, Yagyu 1472–1488.
Combined evaluation of Fibrosis‐4 index and fatty liver for stratifying the risk for diabetes mellitus, Todo 1577–1584.
Impact of the first announced state of emergency owing to coronavirus disease 2019 on stress and blood pressure levels among patients with type 2 diabetes mellitus in Japan, Ito 1607–1616.
HbA1c measurement may save COVID‐19 inpatients from overlooked diabetes, Yoroidaka 1925–1933.
Gaps in the management of diabetes in Asia: A need for improved awareness and strategies in men's sexual health, Kang 1945–1957.
Time trends (2012–2020) in glycated hemoglobin and adherence to the glycemic targets recommended for elderly patients by the Japan Diabetes Society/Japan Geriatrics Society Joint Committee among memory clinic patients with diabetes mellitus, Sugimoto 2038–2046.

Diabetes mellitus, type 1

Reimagining care for young adults living with type 1 diabetes, Morrissey 1294–1299.

Diabetes mellitus type 2

Current status of oral antidiabetic drug prescribing patterns based on the body mass index for Japanese type 2 diabetes mellitus patients and yearly changes in diabetologists' prescribing patterns from 2002 to 2019 (JDDM61), Yagi 65–73.
Low fasting glucose‐to‐estimated average glucose ratio was associated with superior response to insulin degludec/aspart compared with basal insulin in patients with type 2 diabetes, Jang 85–93.
Efficacy and safety of once‐weekly semaglutide in Japanese individuals with type 2 diabetes by baseline age and body mass index, Yabe 1161–1174.
Reduction in cardiovascular disease events in patients with type 2 diabetes mellitus treated with a sodium–glucose cotransporter 2inhibitor versus a dipeptidyl peptidase‐4 inhibitor: A real‐world retrospective administrative database analysis in Japan, Kashiwagi 1175–1189.
Diabetes mellitus, type 2
Analysis of clinical phenotypes of neuropathic symptoms in patients with type 2 diabetes: A multicenter study, Kim 1852–1860.

Diabetes, type 2

Trends of severe hypoglycemia in patients with type 2 diabetes in Korea: A longitudinal nationwide cohort study, Yun 1438–1443.
Adherence to healthy lifestyle behaviors as a preventable risk factor for severe hypoglycemia in people with type 2 diabetes: A longitudinal nationwide cohort study, Yun 1533–1542.

Diabetes prevention

Improving and maintaining healthy lifestyles are associated with a lower risk of diabetes: A large cohort study, Kuwahara 714–724.

Diabetic complication

Comparison of continuous subcutaneous insulin infusion treatment and multiple daily injection treatment on the progression of diabetic complications in Japanese patients with juvenile‐onset type 1 diabetes mellitus, Masuda 1528–1532.

Diabetic complications

Relationship between the early initiation of insulin treatment and diabetic complications inpatients newly diagnosed with type 2 diabetes mellitus in Korea: A nationwide cohort study, Jeon 830–838.

Diabetic foot

When amputation is not the end of the challenge: A successful therapy for osteomyelitis and soft tissue infection in a patient with type 1 diabetes, Caruso 209–212.
Impact of wound microbiology on limb preservation in patients with diabetic foot infection, Hung 336–343.
Association between vitamin D status and diabetic foot in patients with type 2 diabetes mellitus, Tang 1213–1221.

Diabetic foot ulceration

Prevalence and risk factors for diabetic peripheral neuropathy, neuropathic pain and foot ulceration in the Arabian Gulf region, Ponirakis 1551–1559.

Diabetic ketoacidosis

A case of polyneuropathy associated with diabetic ketoacidosis in new‐onset type 1 diabetes, Sada 918–922.
Serious diabetic ketoacidosis induced by insulin allergy and anti‐insulin antibody in an individual with type 2 diabetes mellitus, Takahashi 1788–1792.

Diabetic ketoacidosis with alkalemia

Maturity‐onset diabetes of the young type 5, presenting as diabetic ketoacidosis with alkalemia: A report of a case, Hayakawa‐Iwamoto 923–926.

Diabetic kidney disease

Role of the adaptive immune system in diabetic kidney disease, Kong 213–226.
Synergistic association of the copper/zinc ratio under inflammatory conditions with diabetic kidney disease in patients with type 2 diabetes: The Asahi Diabetes Complications Study, Takao 299–307.
Dyslipidemia in diabetic kidney disease classified by proteinuria and renal dysfunction: A cross‐sectional study from a regional diabetes cohort, Hirano 657–667.

Diabetic macular edema

Treatment of diabetic macular edema in real‐world clinical practice: The effect of aging, Kusuhara 1339–1346.

Diabetic microvascular diseases

Fragmented QRS on electrocardiography as a predictor for diastolic cardiac dysfunction in type 2 diabetes, Yagi 1052–1061.

Diabetic nephropathy

VX‐765 ameliorates renal injury and fibrosis in diabetes by regulating caspase‐1‐mediated pyroptosis and inflammation, Wen 22–33.
Effect of depression on progression to end‐stage renal disease or pre‐end‐stage renal disease death in advanced diabetic nephropathy: A prospective cohort study of the Diabetes Study from the Center of Tokyo Women's Medical University, Horiba 94–101.
Application of weighted gene co‐expression network analysis to identify novel key genes in diabetic nephropathy, Wang 112–124.
Activation of complement C1q and C3 in glomeruli might accelerate the progression of diabetic nephropathy: Evidence from transcriptomic data and renal histopathology, Jiao 839–849.
Correlation of dehydroepiandrosterone with diabetic nephropathy and its clinical value in early detection, Ma 1695–1702.

Diabetic neuropathies

Analysis of clinical phenotypes of neuropathic symptoms in patients with type 2 diabetes: A multicenter study, Kim 1852–1860.

Diabetic neuropathy

Point‐of‐care sural nerve conduction could predict the presence of cardiovascular autonomic neuropathy in type 1 diabetes mellitus, Nattero‐Chávez 1347–1356.
Progressive loss of corneal nerve fibers is associated with physical inactivity and glucose lowering medication associated with weight gain in type 2 diabetes, Ponirakis 1703–1710.

Diabetic peripheral neuropathy

Corneal confocal microscopy for the diagnosis of diabetic peripheral neuropathy: A systematic review and meta‐analysis, Gad 134–147.
Association of glycemic variability assessed by continuous glucose monitoring with subclinical diabetic polyneuropathy in type 2 diabetes patients, Pan 328–335.
Heart rate‐corrected QT interval: A novel diagnostic biomarker for diabetic peripheral neuropathy, Jiang 850–857.
Prevalence and risk factors for diabetic peripheral neuropathy, neuropathic pain and foot ulceration in the Arabian Gulf region, Ponirakis 1551–1559.

Diabetic polyneuropathy

Association between sarcopenia and the severity of diabetic polyneuropathy assessed by nerve conduction studies in Japanese patients with type 2 diabetes mellitus, Mikura 1357–1365.

Diabetic retinopathy

Long non‐coding ribonucleic acid ATP2B1‐AS1modulates endothelial permeability through regulating the miR‐4,729–IQGAP2 axis in diabetic retinopathy, Ren 443–452.
Ocular expression of cyclin‐dependent kinase5 in patients with proliferative diabetic retinopathy, Sano 628–637.
CircZNF532 knockdown protects retinal pigment epithelial cells against high glucose‐induced apoptosis and pyroptosis by regulating the miR‐20b‐5p/STAT3 axis, Liang 781–795.

Diabetic vascular injury

Hyperlipidemic plasma molecules bind and inhibit adiponectin activity, Zhang 947–954.

Diagnosis

Corneal confocal microscopy for the diagnosis of diabetic peripheral neuropathy: A systematic review and meta‐analysis, Gad 134–147.
Heart rate‐corrected QT interval: A novel diagnostic biomarker for diabetic peripheral neuropathy, Jiang 850–857.

Diarrhea

Effects of probiotic *Bifidobacterium bifidum* G9‐1 on the gastrointestinal symptoms of patients with type 2 diabetes mellitus treated with metformin: An open‐label, single‐arm, exploratory research trial, Hata 489–500.

Diastolic cardiac dysfunction

Fragmented QRS on electrocardiography as a predictor for diastolic cardiac dysfunction in type 2 diabetes, Yagi 1052–1061.

Dietary carbohydrates

Dietitian‐supported dietary intervention leads to favorable dietary changes in patients with type 2 diabetes: A randomized controlled trial, Kawabata 1963–1970.

Dietary services

Dietitian‐supported dietary intervention leads to favorable dietary changes in patients with type 2 diabetes: A randomized controlled trial, Kawabata 1963–1970.

Dipeptidylpeptidase‐4 inhibitor

Dipeptidyl peptidase‐4 inhibitor and insulin combination treatment in type 2 diabetes and chronic kidney disease: A meta‐analysis, Zhou 468–477.

Dipeptidyl peptidase‐4 inhibitors

Association between dipeptidyl peptidase‐4 inhibitors and increased risk for bullous pemphigoid within 3 months from first use: A 5‐year population‐based cohort study using the Japanese National Database, Kuwata 460–467.

Disability

Effect of diabetes and prediabetes on the development of disability and mortality among middle‐aged Japanese adults: A 22‐year follow up of NIPPON DATA 90, Hoang 1897–1904.

DNA methylation

Novel method utilizing bisulfite conversion with dual amplification‐refractory mutation system polymerase chain reaction to detect circulating pancreatic β‐cell cfDNA, Okada 1140–1148.

Dropout

Factors associated with the degree of glycemic deterioration among patients with type 2 diabetes who dropped out of diabetes care: A longitudinal analysis using medical claims and health checkup data in Japan, Ihana‐Sugiyama 571–579.

Dulaglutide

The renoprotective effect of once‐weekly GLP‐1 receptor agonist dulaglutide on progression of nephropathy in Japanese patients with type 2 diabetes and moderate to severe chronic kidney disease (JDDM67), Tsuchida 1834–1841.

Editorial

Revisiting the strategies for the pharmacological management of type 2 diabetes – From glycemic control, organ protection, safety to weight reduction, Li 3–5
Not out of the woods yet: “Diabetic neuropathy” or “neuropathy associated with diabetes”?, Himeno 753–755.
Evolution and diabetic vasculopathy, Yamamoto 1111–1113.
Diabetes care in China: Innovations and implications, Jia 1795–1797.

Eicosapentaenoic acid

Effects of eicosapentaenoic acid on serum levels of selenoprotein P and organ‐specific insulin sensitivity in humans with dyslipidemia and type 2 diabetes, Takeshita 532–542.

Elderly diabetes patient

Clinical features and sulfonylurea usage among outpatients with diabetes aged ≥90 years in an urban diabetes clinic in Tokyo, Kobori 2010–2017.

Elderly patients

β‐Cell function and body mass index are predictors of exercise response in elderly patients with prediabetes, He 1253–1261.

Elderly patients with diabetes

Voxel‐based specific regional analysis system for Alzheimer's disease utility as a screening tool for unrecognized cognitive dysfunction of elderly patients in diabetes outpatient clinics: Multicenter retrospective exploratory study, Ueba 177–184.

Electrochemical skin conductance

The early diagnostic value of ankle‐brachial index combined with feet electrochemical skin conductance for peripheral artery disease in type 2 diabetes, Zhao 525–531.

End‐stage renal disease

Effect of depression on progression to end‐stage renal disease or pre‐end‐stage renal disease death in advanced diabetic nephropathy: A prospective cohort study of the Diabetes Study from the Center of Tokyo Women's Medical University, Horiba 94–101.

Energy intake

Dietitian‐supported dietary intervention leads to favorable dietary changes in patients with type 2 diabetes: A randomized controlled trial, Kawabata 1963–1970.

Enterovirus

Immunohistochemical detection of enteroviruses in pancreatic tissues of patients with type 1 diabetes using a polyclonal antibody against 2A protease of Coxsackievirus, Jimbo 435–442.

Epidemiology

Association between fat mass index, fat‐free mass index and hemoglobin A1c in a Japanese population: The Tohoku Medical Megabank Community‐based Cohort Study, Takase 858–867.

Ertugliflozin

Effectiveness and safety of ertugliflozin for type 2 diabetes: A meta‐analysis of data from randomized controlled trials, Zhang 478–488.

Esaxerenone

Reduction in the magnitude of serum potassium elevation in combination therapy with esaxerenone (CS‐3150) and sodium–glucose cotransporter 2 inhibitor in patients with diabetic kidney disease: Subanalysis of two phase III studies, Shikata 1190–1202.

Estimated glomerular filtration rate

Relationship of coffee consumption with a decline in kidney function among patients with type 2 diabetes: The Fukuoka Diabetes Registry, Komorita 1030–1038.
The renoprotective effect of once‐weekly GLP‐1 receptor agonist dulaglutide on progression of nephropathy in Japanese patients with type 2 diabetes and moderate to severe chronic kidney disease (JDDM67), Tsuchida 1834–1841.

Exercise response

β‐Cell function and body mass index are predictors of exercise response in elderly patients with prediabetes, He 1253–1261.

Exocytosis

Direct visualization of glucagon‐like peptide‐1secretion by fluorescent fusion proteins, Tsuzuki 1134–1139.

Fasting

Use of automated insulin delivery systems in people with type 1 diabetes fasting during Ramadan: An observational study, Aldibbiat 647–651.

Fasting plasma glucose level

Fasting plasma glucose level in the range of 90–99 mg/dL and the risk of the onset of type 2 diabetes: Population‐based Panasonic cohort study 2, Munekawa 453–459.

Fat

Glucose‐dependent insulinotropic polypeptide secretion after oral macronutrient ingestion: The human literature revisited and a systematic study in model experiments in mice, Ahrén 1655–1665.

Fat distribution

Effect of glucagon‐like peptide‐1 receptor agonists on fat distribution in patients with type 2 diabetes: A systematic review and meta‐analysis, Duan 1149–1160.

Fatty liver

Metabolic associated fatty liver disease is a risk factor for chronic kidney disease, Hashimoto 308–316.
Fatty liver with metabolic disorder, such as metabolic dysfunction‐associated fatty liver disease, indicates high risk for developing diabetes mellitus, Miyake 1245–1252.

Fatty liver index

Circulating level of fatty acid‐binding protein 4is an independent predictor of metabolic dysfunction‐associated fatty liver disease in middle‐aged and elderly individuals, Tanaka 878–888.

Fetal DNA

Precision medicine in diabetes: A non‐invasive prenatal diagnostic test for the determination of fetal glucokinase mutations, Nouspikel 256–261.

Fibroblast growth factor 21

The relationship between serum adipokine fibroblast growth factor‐21 and gestational diabetes mellitus, Jia 2047–2053.

Fibrosis

Combined evaluation of Fibrosis‐4 index and fatty liver for stratifying the risk for diabetes mellitus, Todo 1577–1584.

Fibrosis‐4 index

Abdominal aortic calcification is associated with Fibrosis‐4 index and low body mass index in type 2 diabetes patients: A retrospective cross‐sectional study, Togashi 1861–1872.

First‐line therapy

Retrospective nationwide study on the trends in first‐line antidiabetic medication for patients with type 2 diabetes in Japan, Bouchi 280–291.

Fixed‐ratio combination

Comparisons of efficacy and safety in insulin glargine and lixisenatide plus glulisine combination therapy with multiple daily injection therapy in Japanese patients with type 2 diabetes, Kawaguchi 505–514.

Flash glucose monitoring

Frequent scanning using flash glucose monitoring contributes to better glycemic control in children and adolescents with type 1 diabetes, Urakami 185–190.
Impact of flash glucose monitoring on glycemic control varies with the age and residual β‐cell function of patients with type 1 diabetes mellitus, Zhang 552–559.

Flow‐mediated vasodilation

Relationship between hemoglobin A1c level and flow‐mediated vasodilation in patients with type 2 diabetes mellitus receiving antidiabetic drugs, Yamaji 677–686.

Foveal avascular zone

Measuring the foveal avascular zone in diabetes: A study using optical coherence tomography angiography, Aitchison 668–676.

Fracture

Sodium–glucose cotransporter 2 inhibitors do not increase the risk of fractures in real‐world clinical practice in Korea: A national observational cohort study, Ha 986–996.

Fragmented QRS

Fragmented QRS on electrocardiography as a predictor for diastolic cardiac dysfunction in type 2 diabetes, Yagi 1052–1061.

Fulminant type 1 diabetes

New‐onset fulminant type 1 diabetes after severe acute respiratory syndrome coronavirus 2 vaccination: A case report, Sasaki 1286–1289.
Two cases with fulminant type 1 diabetes that developed long after cessation of immune checkpoint inhibitor treatment, Hatayama 1458–1460.

Fulminant type 1 diabetes mellitus

Prevalence and clinical characteristics of fulminant type 1 diabetes mellitus in Korean adults: A multi‐institutional joint research, Song 47–53.

Functional balance

Longitudinal effects of one‐leg standing time on neuropathy outcomes in association with glycemic control in non‐elderly patients with type 2 diabetes, Sugimoto 1039–1051.

Gamma‐glutamyl transferase to high‐density lipoprotein cholesterol ratio

Elevated gamma‐glutamyl transferase to high‐density lipoprotein cholesterol ratio has a non‐linear association with incident diabetes mellitus: A second analysis of a cohort study, Hu 2027–2037.

Gastric residue,

Association of insulin treatment with gastric residue during an esophagogastroduo‐denoscopy, Kobori 501–504.

Gastroparesis

Association of insulin treatment with gastric residue during an esophagogastroduo‐denoscopy, Kobori 501–504.

Generalized lipodystrophy‐associated progeroid syndrome

Severe aortic stenosis during leptin replacement therapy in a patient with generalized lipodystrophy‐associated progeroid syndrome due to an *LMNA* variant: A case report, Sasako 1636–1638.

Geriatric nutritional risk index

Sarcopenia is associated with the Geriatric Nutritional Risk Index in elderly patients with poorly controlled type 2 diabetes mellitus, Matsuura 1366–1373.

Gestational diabetes

Plasma cytokines during pregnancy provide insight into the risk of diabetes in the gestational diabetes risk group, Tagoma 1596–1606.

Gestational diabetes mellitus

Neurodevelopmental delay up to the age of 4 years in infants born to women with gestational diabetes mellitus: The Japan Environment and Children's Study, Saito 2054–2062.
The association between weight gain at different stages of pregnancy and risk of gestational diabetes mellitus, Chuang 359–366.
Association between pre‐pregnancy body mass index and gestational weight gain and perinatal outcomes in pregnant women diagnosed with gestational diabetes mellitus: The Japan Environment and Children's Study, Saito 889–899.
Maternal sleep during pregnancy and adverse pregnancy outcomes: A systematic review and meta‐analysis, Wang 1262–1276.
Comparison of the clinical effects of intermittently scanned and real‐time continuous glucose monitoring in children and adolescents with type 1 diabetes: A retrospective cohort study, Urakami 1745–1752.
The relationship between serum adipokine fibroblast growth factor‐21 and gestational diabetes mellitus, Jia 2047–2053.

Gestational weight gain

The association between weight gain at different stages of pregnancy and risk of gestational diabetes mellitus, Chuang 359–366.
Association between pre‐pregnancy body mass index and gestational weight gain and perinatal outcomes in pregnant women diagnosed with gestational diabetes mellitus: The Japan Environment and Children's Study, Saito 889–899.

Glomerular hyperfiltration

Adenosine/adenosine type 1 receptor signaling pathway did not play dominant roles on the influence of sodium–glucose cotransporter 2 inhibitor in the kidney of bovine serum albumin‐overloaded streptozotocin‐induced diabetic mice, Shimada 955–964.

Glomeruli

Activation of complement C1q and C3 in glomeruli might accelerate the progression of diabetic nephropathy: Evidence from transcriptomic data and renal histopathology, Jiao 839–849.

Glucagon

Neuronal regulation of glucagon secretion and gluconeogenesis, Thorens 599–607.

Glucagon‐like peptide‐1

Roles of glucose‐dependent insulinotropic polypeptide in diet‐induced obesity, Seino 1122–1128.
Direct visualization of glucagon‐like peptide‐1secretion by fluorescent fusion proteins, Tsuzuki 1134–1139.
Effect of glucagon‐like peptide‐1 receptor agonists on fat distribution in patients with type 2 diabetes: A systematic review and meta‐analysis, Duan 1149–1160.
Efficacy and safety of once‐weekly semaglutide in Japanese individuals with type 2 diabetes by baseline age and body mass index, Yabe 1161–1174.
A randomized trial to investigate the efficacy and safety of once‐daily liraglutide 1.8 mg in Japanese adults with type 2 diabetes exhibiting an inadequate response to liraglutide 0.9 mg, Seino 1321–1329.

Glucagon‐like peptide‐1 analog

Efficacy and safety of oral semaglutide in Japanese patients with type 2 diabetes: A subgroup analysis by baseline variables in the PIONEER 9 and PIONEER 10 trials, Yabe 975–985.

Glucagon‐like peptide‐1 receptor

Efficacy and safety of once‐weekly semaglutide in Japanese individuals with type 2 diabetes in the SUSTAIN 1, 2, 5 and 9 trials: Post‐hoc analysis, Araki 1971–1980.

Glucagon‐like peptide‐1 receptor agonists

Insulin–glucagon‐like peptide‐1 receptor agonist relay and glucagon‐like peptide‐1 receptor agonist first regimens in individuals with type 2 diabetes: A randomized, open‐label trial study, Takeshita 965–974.

Glucose

Glucose‐dependent insulinotropic polypeptide secretion after oral macronutrient ingestion: The human literature revisited and a systematic study in model experiments in mice, Ahrén 1655–1665.

Glucose control

Effects of whole grain intake on glycemic control: A meta‐analysis of randomized controlled trials, Li 1814–1824.

Glucose‐dependent insulinotropic polypeptide

Roles of glucose‐dependent insulinotropic polypeptide in diet‐induced obesity, Seino 1122–1128.
Glucose‐dependent insulinotropic polypeptide secretion after oral macronutrient ingestion: The human literature revisited and a systematic study in model experiments in mice, Ahrén 1655–1665.

Glucose metabolism

miR‐4,431 targets TRIP10/PRKD1 and impairs glucose metabolism, Pan 617–627.

Glucose tolerance

Metabolic state switches between morning and evening in association with circadian clock in people without diabetes, Fujimoto 1496–1505.

Glucose toxicity

Insulin–glucagon‐like peptide‐1 receptor agonist relay and glucagon‐like peptide‐1 receptor agonist first regimens in individuals with type 2 diabetes: A randomized, open‐label trial study, Takeshita 965–974.

Glucose transporter

Placental expression of glucose transportersGLUT‐1, GLUT‐3, GLUT‐8 and GLUT‐12 in pregnancies complicated by gestational and type 1 diabetes mellitus, Stanirowski 560–570.

Glycated hemoglobin

Diabetes management by either telemedicine or clinic visit improved glycemic control during the coronavirus disease 2019 pandemic state of emergency in Japan, Onishi 386–390.
Association between fat mass index, fat‐free mass index and hemoglobin A1c in a Japanese population: The Tohoku Medical Megabank Community‐based Cohort Study, Takase 858–867.
HbA1c level may be a risk factor for oxygen therapy requirement in patients with coronavirus disease 2019, Yanagisawa 909–917.
Current status of diabetes treatment in Miyazaki Prefecture, Japan: Results of a questionnaire survey conducted in 2016 and 2020, Uehira 1011–1020.
Discordantly high glycated hemoglobin might assist in diagnosing α‐thalassemia, but not diabetes: A case report, Gao 1633–1635.
Glycated hemoglobin level on admission associated with progression to severe disease in hospitalized patients with non‐severe coronavirus disease 2019, Numaguchi 1779–1787.
Efficacy and safety of once‐weekly semaglutide in Japanese individuals with type 2 diabetes in the SUSTAIN 1, 2, 5 and 9 trials: Post‐hoc analysis, Araki 1971–1980.
Time trends (2012–2020) in glycated hemoglobin and adherence to the glycemic targets recommended for elderly patients by the Japan Diabetes Society/Japan Geriatrics Society Joint Committee among memory clinic patients with diabetes mellitus, Sugimoto 2038–2046.

Glycated hemoglobin A1c

HbA1c measurement may save COVID‐19 inpatients from overlooked diabetes, Yoroidaka 1925–1933.

Glycemic control

Factors associated with the degree of glycemic deterioration among patients with type 2 diabetes who dropped out of diabetes care: A longitudinal analysis using medical claims and health checkup data in Japan, Ihana‐Sugiyama 571–579.
Lifestyle changes and their impact on glycemic control and weight control in patients with diabetes during the coronavirus disease 2019 pandemic in Japan, Takahara 375–385.
Efficacy and safety of oral semaglutide in Japanese patients with type 2 diabetes: A subgroup analysis by baseline variables in the PIONEER 9 and PIONEER 10 trials, Yabe 975–985.
Living and working environments are important determinants of glycemic control in patients with diabetes during the COVID‐19 pandemic: A retrospective observational study, Terakawa, 1094–1104.
Factors associated with hypoglycemia unawareness and severe hypoglycemia in type 1 diabetes mellitus patients, Takagi 2018–2026.
Time trends (2012–2020) in glycated hemoglobin and adherence to the glycemic targets recommended for elderly patients by the Japan Diabetes Society/Japan Geriatrics Society Joint Committee among memory clinic patients with diabetes mellitus, Sugimoto 2038–2046.

Glycemic exposure

Longitudinal effects of one‐leg standing time on neuropathy outcomes in association with glycemic control in non‐elderly patients with type 2 diabetes, Sugimoto 1039–1051.

Glycemic variability

Association of glycemic variability assessed by continuous glucose monitoring with subclinical diabetic polyneuropathy in type 2 diabetes patients, Pan 328–335.

Glycosylated hemoglobin

Measuring the foveal avascular zone in diabetes: A study using optical coherence tomography angiography, Aitchison 668–676.

Gram‐negative bacteria.

Impact of wound microbiology on limb preservation in patients with diabetic foot infection, Hung 336–343.

GSDMD

VX‐765 ameliorates renal injury and fibrosis in diabetes by regulating caspase‐1‐mediated pyroptosis and inflammation, Wen 22–33.

Handgrip strength

Lower handgrip strength levels probably precede triglyceride glucose index and associated with diabetes in men not in women, Zheng 148–155.
Poor glycemic control rather than types of diabetes is a risk factor for sarcopenia in diabetes mellitus: The MUSCLES‐DM study, Hiromine 1881–1888.

Health resources

Healthcare resource utilization in patients treated with empagliflozin in East Asia, Sheu 810–821.

Healthy lifestyle

Impact of healthy lifestyle on the risk of type 2 diabetes mellitus in southwest China: A prospective cohort study, Wu 2091–210.

Hearing impairment

Impact of vision and hearing impairments on risk of cardiovascular outcomes and mortality in patients with type 2 diabetes: A nationwide cohort study, Jung 515–524.

Heart failure

Efficacy and safety of once‐weekly semaglutide in Japanese individuals with type 2 diabetes by baseline age and body mass index, Yabe 1161–1174.

Heart rate recovery

Delayed heart rate recovery after exercise predicts development of metabolic syndrome: A retrospective cohort study, Yu 167–176.

Heart rate variability

Utility of using electrocardiogram measures of heart rate variability as a measure of cardiovascular autonomic neuropathy in type 1diabetes patients, Pop‐Busui 125–133.

Hemoglobin A1c

Comparison of the effects of frequent versus conventional nutritional interventions inpatients with type 2 diabetes mellitus: A randomized, controlled trial, Kawabata 271–279.
Relationship between hemoglobin A1c level and flow‐mediated vasodilation in patients with type 2 diabetes mellitus receiving antidiabetic drugs, Yamaji 677–686.

Hepatokine

Circulating level of fatty acid‐binding protein 4is an independent predictor of metabolic dysfunction‐associated fatty liver disease in middle‐aged and elderly individuals, Tanaka 878–888.

High‐density lipoprotein cholesterol

Cholesterol efflux capacity of high‐density lipoprotein was not associated with cognitive decline and brain structures in older people with diabetes mellitus, Wu 1873–1880.

High mobility group AT‐hook 2

Circular ribonucleic acid nucleoporin 98 knockdown alleviates high glucose‐induced proliferation, fibrosis, inflammation and oxidative stress in human glomerular mesangial cells by regulating the microribonucleic acid‐151‐3p–high mobility group AT‐hook 2 axis, Dong 1303–1315.

High‐performance liquid chromatography

Discordantly high glycated hemoglobin might assist in diagnosing α‐thalassemia, but not diabetes: A case report, Gao 1633–1635.

HNF1B

Maturity‐onset diabetes of the young type 5, presenting as diabetic ketoacidosis with alkalemia: A report of a case, Hayakawa‐Iwamoto 923–926.

Home isolation

Implementation of diabetes care and educational program via telemedicine inpatients with COVID‐19 in home isolation in Thailand: A real‐world experience, Harindhanavudhi 1448–1457.

Homeostasis model assessment

Usefulness of subclassification of adult diabetes mellitus among inpatients in Japan, Saito 706–713.

Hospitalization

Clinical course of adolescents with type 2 diabetes mellitus: A nationwide cohort study in Taiwan, Yen 1905–1913.

Humans

Glucose‐dependent insulinotropic polypeptide secretion after oral macronutrient ingestion: The human literature revisited and a systematic study in model experiments in mice, Ahrén 1655–1665.

Hyperglycemia

Association of glycated hemoglobin at an early stage of pregnancy with the risk of gestational diabetes mellitus among non‐diabetic women in Japan: The Japan Environment and Children's Study, Sekine 687–695.
Diet‐induced prediabetes: Effects on the activity of the renin–angiotensin–aldosterone system in selected organs, Mkhize 768–780.
Impact of newly diagnosed diabetes on coronavirus disease 2019 severity and hyperglycemia, Uchihara 1086–1093.

Hyperlipidemia

Hyperlipidemic plasma molecules bind and inhibit adiponectin activity, Zhang 947–954.

Hypertriglyceridemia

Maternally inherited diabetes and deafness coexists with lipoprotein lipase gene mutation‐associated severe hyperlipidemia that was resistant to fenofibrate and atorvastatin, but sensitive to bezafibrate: A case report, Zhang 397–401.

Hypoglycemia

Neuronal regulation of glucagon secretion and gluconeogenesis, Thorens 599–607.
Comparison of the clinical effects of intermittently scanned and real‐time continuous glucose monitoring in children and adolescents with type 1 diabetes: A retrospective cohort study, Urakami 1745–1752.

Hypoglycemic agent

Current status of diabetes treatment in Miyazaki Prefecture, Japan: Results of a questionnaire survey conducted in 2016 and 2020, Uehira 1011–1020.

Hypoglycemia unawareness

High prevalence of depressive symptoms among people with pediatric‐onset and adolescent‐onset type 1 diabetes: A cross‐sectional analysis of the Diabetes Study from the Center of Tokyo Women's Medical University, Takaike 1626–1632.

Hypothalamus

Neuronal regulation of glucagon secretion and gluconeogenesis, Thorens 599–607.

Ideal cardiovascular health metrics

Association of ideal cardiovascular health metrics and incident type 2 diabetes mellitus among an urban population of Iran: One decade follow up in the Tehran Lipid and Glucose Study, Asgari 1711–1722.

IDegLira

Preserved pharmacokinetics and pharmacodynamics of insulin degludec and liraglutide when administered as insulin degludec/liraglutide in a Chinese population, Liu 652–656.

Imeglimin

A novel mechanism of imeglimin‐mediated insulin secretion via the cADPR‐TRP channel pathway, Funazaki 34–41.

Immune checkpoint inhibitors

Two cases with fulminant type 1 diabetes that developed long after cessation of immune checkpoint inhibitor treatment, Hatayama 1458–1460.

Immune‐related adverse events

Two cases with fulminant type 1 diabetes that developed long after cessation of immune checkpoint inhibitor treatment, Hatayama 1458–1460.

Immunosuppression therapy

A unique profile of insulin antibody titer in islet‐transplanted patients, Keidai 1939–1942.

Impaired fasting glucose

Effects of multi‐metal exposure on the risk of diabetes mellitus among people aged 40–75 years in rural areas in southwest China, Zhang 1412–1425.

Impaired glucose tolerance

Cardiovascular events and atherosclerosis inpatients with type 2 diabetes and impaired glucose tolerance: What are the medical treatments to prevent cardiovascular events in such patients?, Ishii 1114–1121.

Incidence

Impact of healthy lifestyle on the risk of type 2 diabetes mellitus in southwest China: A prospective cohort study, Wu 2091–210.

Incident metabolic syndrome

Delayed heart rate recovery after exercise predicts development of metabolic syndrome: A retrospective cohort study, Yu 167–176.

Inflammation

VX‐765 ameliorates renal injury and fibrosis in diabetes by regulating caspase‐1‐mediated pyroptosis and inflammation, Wen 22–33.
Role of the adaptive immune system in diabetic kidney disease, Kong 213–226.

Insulin

Mitochondrial haplogroups have a better correlation to insulin requirement than nuclear genetic variants for type 2 diabetes mellitus in Taiwanese individuals, Shen 201–208.
Relationship between the early initiation of insulin treatment and diabetic complications inpatients newly diagnosed with type 2 diabetes mellitus in Korea: A nationwide cohort study, Jeon 830–838.
Efficacy and safety of sitagliptin and insulin for latent autoimmune diabetes in adults: A systematic review and meta‐analysis, Lin 1506–1519.

Insulin allergy

Serious diabetic ketoacidosis induced by insulin allergy and anti‐insulin antibody in an individual with type 2 diabetes mellitus, Takahashi 1788–1792.

Insulin antibodies

A unique profile of insulin antibody titer in islet‐transplanted patients, Keidai 1939–1942.

Insulin degludec/insulin aspart

Low fasting glucose‐to‐estimated average glucose ratio was associated with superior response to insulin degludec/aspart compared with basal insulin in patients with type 2 diabetes, Jang 85–93.

Insulin–glucagon‐like peptide‐1 receptor agonist relay regimen

Insulin–glucagon‐like peptide‐1 receptor agonist relay and glucagon‐like peptide‐1receptor agonist first regimens in individuals with type 2 diabetes: A randomized, open‐label trial study, Takeshita 965–974.

Insulin resistance

Lower handgrip strength levels probably precede triglyceride glucose index and associated with diabetes in men not in women, Zheng 148–155.
Oral fat tolerance testing identifies abnormal pancreatic β‐cell function and insulin resistance in individuals with normal glucose tolerance, Liu 1805–1813.
Time in range assessed by capillary blood glucose in relation to insulin sensitivity and β‐cell function in patients with type 2 diabetes mellitus: A cross‐sectional study in China, Ye 1825–1833.

Insulin secretion

Perioperative C‐peptide index is associated with the status of diabetes management after pancreatectomy, Shikata 1685–1694.
Oral fat tolerance testing identifies abnormal pancreatic β‐cell function and insulin resistance in individuals with normal glucose tolerance, Liu 1805–1813.

Insulin secretion and sensitivity

Metabolic state switches between morning and evening in association with circadian clock in people without diabetes, Fujimoto 1496–1505.

Insulin sensitivity

Effects of whole grain intake on glycemic control: A meta‐analysis of randomized controlled trials, Li 1814–1824.

Insulin treatment

Association of insulin treatment with gastric residue during an esophagogastroduo‐denoscopy, Kobori 501–504.
Concomitant treatment with insulin and sodium–glucose cotransporter 2 inhibitors was associated with the renal composite outcome in Japanese patients with type 2 diabetes and chronic kidney disease: A propensity score‐matched analysis, Toyoda 1520–1527.

Interleukin‐6

Development of type 1 diabetes in a patient treated with anti‐interleukin‐6 receptor antibody for rheumatoid arthritis, Kawasaki 738–740.

Intermittent very‐low calorie diet.

Effects of intermittent very‐low calorie diet on glycemic control and cardiovascular risk factors in obese patients with type 2 diabetes mellitus: A randomized controlled trial, Umphonsathien 156–166.

International Association of Diabetes and Pregnancy Study Groups.

Comparison of the clinical effects of intermittently scanned and real‐time continuous glucose monitoring in children and adolescents with type 1 diabetes: A retrospective cohort study, \ Urakami 1745–1752.

Islet pathology

Diversity of pathophysiology in type 2 diabetes shown by islet pathology, Mizukami 6–13.

Islet transplantation

A unique profile of insulin antibody titer in islet‐transplanted patients, Keidai 1939–1942.

JDI Updates

One year into the clash of pandemics of diabetes and COVID‐19: Lessons learnt and future perspectives, Wai Lui 19–21.
Impaired insulin secretion and related factors in East Asian individuals, Nakamura 233–235.
Updates in diabetic neuropathy: A call for new diagnostic and treatment approaches, Aso 432–434.
Cardiovascular disease in patients with type 2 diabetes, Jeon 614–616.
A new era of diabetic kidney disease treatment with sodium–glucose cotransporter‐2 inhibitors, Maegawa 765–767.
Sarcopenia in elderly diabetes, Jiang 944–946.
Updates for hyperglycemia in pregnancy: The ongoing journey for maternal–neonatal health, Li 1652–1654.
Current status of diabetic kidney disease and latest trends in management, Kim 1961–1962.
Urinary albumin‐to‐creatinine ratio.
Metabolic Score for Insulin Resistance, a novel score to evaluate insulin sensitivity, is associated with the urinary albumin‐to‐creatinine ratio in Chinese adults: A cross‐sectional REACTION study, Su 1222–1234.
Correlation of dehydroepiandrosterone with diabetic nephropathy and its clinical value in early detection, Ma 1695–1702.

Ketoacidosis

Type 1 diabetes mellitus following COVID‐19 RNA‐based vaccine, Sakurai 1290–1292.

Kidney disease

Metabolic associated fatty liver disease is a risk factor for chronic kidney disease, Hashimoto 308–316.

Korea

Prevalence and clinical characteristics of fulminant type 1 diabetes mellitus in Korean adults: A multi‐institutional joint research, Song 47–53.

Laparoscopic sleeve gastrectomy

Therapeutic effect of laparoscopic sleeve gastrectomy on obstructive sleep apnea and relationship of type 2 diabetes in Japanese patients with severe obesity, Yanari 1073–1085.

Latent autoimmune diabetes in adults

Efficacy and safety of sitagliptin and insulin for latent autoimmune diabetes in adults: A systematic review and meta‐analysis, Lin 1506–1519.

Late‐stage elderly

Current status of low‐density lipoprotein cholesterol for primary prevention of coronary artery disease in late‐stage elderly persons with type 2 diabetes mellitus: A retrospective, single‐center study, Yamamoto 1567–1576.

Leptin replacement therapy

Severe aortic stenosis during leptin replacement therapy in a patient with generalized lipodystrophy‐associated progeroid syndrome due to an *LMNA* variant: A case report, Sasako 1636–1638.

Letter to the Editor

Diabetic ketoacidosis and high mortality among patients with coronavirus disease 2019 in a Peruvian hospital, Lopez‐Huamanrayme 746–748.
Comment on a recent article titled “Circulating level of fatty acid‐binding protein 4 is an independent predictor of metabolic dysfunction‐associated fatty liver disease in middle‐aged and elderly individuals”, Naharci 927.
Reply to the comments of Naharci et al. on “Circulating level of fatty acid‐binding protein 4 is an independent predictor of metabolic dysfunction‐associated fatty liver disease in middle‐aged and elderly individuals”, Tanaka 928–929.
Immune response to the severe acute respiratory syndrome coronavirus 2 vaccines: Is it sustained in the diabetes population?, Rouka 1461–1462.
A pitfall in hemoglobin A1c measurement with high performance liquid chromatography method in the diagnosis of onset of fulminant type 1 diabetes mellitus, Oe 1943–1944.
Onset of type 1 diabetes during bone growth period is associated with increased prevalence of bone fracture: A post‐hoc analysis of a cross‐sectional study, Komorita 2101–2102.

Leukocyte counts

Association of leukocyte counts in the first trimester with glucose intolerance during pregnancy, Wang 191–200.

Lifestyle behavior

Adherence to healthy lifestyle behaviors as a preventable risk factor for severe hypoglycemia in people with type 2 diabetes: A longitudinal nationwide cohort study, Yun 1533–1542.

Lifestyle change

Lifestyle changes and their impact on glycemic control and weight control in patients with diabetes during the coronavirus disease 2019 pandemic in Japan, Takahara 375–385.

Lifestyle changes

Living and working environments are important determinants of glycemic control in patients with diabetes during the COVID‐19 pandemic: A retrospective observational study, Terakawa, 1094–1104.

Lipohypertrophy

Repeated insulin injection without site rotation affects skin thickness – ultrasonographic and histological evaluation, Murao 997–1003.

Lipoprotein lipase

Maternally inherited diabetes and deafness coexists with lipoprotein lipase gene mutation‐associated severe hyperlipidemia that was resistant to fenofibrate and atorvastatin, but sensitive to bezafibrate: A case report, Zhang 397–401.

Liraglutide

A randomized trial to investigate the efficacy and safety of once‐daily liraglutide 1.8 mg in Japanese adults with type 2 diabetes exhibiting an inadequate response to liraglutide 0.9 mg, Seino 1321–1329.

Longitudinal cohort study

Delayed heart rate recovery after exercise predicts development of metabolic syndrome: A retrospective cohort study, Yu 167–176.

Low‐density lipoprotein cholesterol

Current status of low‐density lipoprotein cholesterol for primary prevention of coronary artery disease in late‐stage elderly persons with type 2 diabetes mellitus: A retrospective, single‐center study, Yamamoto 1567–1576.

Low‐density lipoprotein triglycerides

Dyslipidemia in diabetic kidney disease classified by proteinuria and renal dysfunction: A cross‐sectional study from a regional diabetes cohort, Hirano 657–667.

Lower‐extremities amputation

Impact of wound microbiology on limb preservation in patients with diabetic foot infection, Hung 336–343.

Lower‐limb amputation

Real‐world risk of lower‐limb amputation associated with sodium–glucose cotransporter 2 inhibitors versus metformin: A propensity score‐matched model analysis in Japan, Mizutani 2000–2009.

Machine learning

Predictive ability of current machine learning algorithms for type 2 diabetes mellitus: A meta‐analysis, Kodama 900–908.
Non‐laboratory‐based risk assessment model for case detection of diabetes mellitus and pre‐diabetes in primary care, Dong 1374–1386.

Macronutrients

Glucose‐dependent insulinotropic polypeptide secretion after oral macronutrient ingestion: The human literature revisited and a systematic study in model experiments in mice, Ahrén 1655–1665.

Macrovascular complications

Relationship between onset age of type 2 diabetes mellitus and vascular complications based on propensity score matching analysis, Huang 1062–1072.

Magnetic resonance imaging

Voxel‐based specific regional analysis system for Alzheimer's disease utility as a screening tool for unrecognized cognitive dysfunction of elderly patients in diabetes outpatient clinics: Multicenter retrospective exploratory study, Ueba 177–184.

Male hypogonadism

Gaps in the management of diabetes in Asia: A need for improved awareness and strategies in men's sexual health, Kang 1945–1957.

Management

HbA1c, blood pressure, and cholesterol control in adults with diabetes: A report card for Kuwait, Alkandari 1732–1739.

Maternally inherited diabetes and deafness

Maternally inherited diabetes and deafness coexists with lipoprotein lipase gene mutation‐associated severe hyperlipidemia that was resistant to fenofibrate and atorvastatin, but sensitive to bezafibrate: A case report, Zhang 397–401.

Maturity‐onset diabetes of the young

Precision diabetes: Lessons learned from maturity‐onset diabetes of the young (MODY), Tosur 465–1471.

Maturity‐onset diabetes of the young type 5

Maturity‐onset diabetes of the young type 5, presenting as diabetic ketoacidosis with alkalemia: A report of a case, Hayakawa‐Iwamoto 923–926.

Meta‐analysis

Efficacy of acceptance and commitment therapy for people with type 2 diabetes: Systematic review and meta‐analysis, Sakamoto 262–270.
Effectiveness and safety of ertugliflozin for type 2 diabetes: A meta‐analysis of data from randomized controlled trials, Zhang 478–488.
Association between visceral adiposity index and risk of prediabetes: A meta‐analysis of observational studies, Wang 543–551.
Predictive ability of current machine learning algorithms for type 2 diabetes mellitus: A meta‐analysis, Kodama 900–908.
Effects of whole grain intake on glycemic control: A meta‐analysis of randomized controlled trials, Li 1814–1824.

Metabolic disease

Fatty liver with metabolic disorder, such as metabolic dysfunction‐associated fatty liver disease, indicates high risk for developing diabetes mellitus, Miyake 1245–1252.

Metabolic disorder

Micro ribonucleic acid‐363 regulates the phosphatidylinositol 3‐kinase/threonine protein kinase axis by targeting NOTCH1 and forkhead box C2, leading to hepatic glucose and lipids metabolism disorder in type 2 diabetes mellitus, Peng 236–248.

Metabolic dysfunction‐associated fatty liver disease

Non‐alcoholic fatty liver disease and type 2diabetes: An update, Lee 930–940.

Metabolic score for insulin resistance

Metabolic Score for Insulin Resistance, a novel score to evaluate insulin sensitivity, is associated with the urinary albumin‐to‐creatinine ratio in Chinese adults: A cross‐sectional REACTION study, Su 1222–1234.

Metabolic syndrome

Metabolic associated fatty liver disease is a risk factor for chronic kidney disease, Hashimoto 308–316.
Association between maternal gestational diabetes mellitus and high‐sensitivity C‐reactive protein levels in 8‐year‐old children: The Yamanashi Adjunct Study of the Japan Environment and Children's Study(JECS), Sekine 1444–1447.

Metabolism

Serotonin in the regulation of systemic energy metabolism, Moon 1639–1645.

Mice

Glucose‐dependent insulinotropic polypeptide secretion after oral macronutrient ingestion: The human literature revisited and a systematic study in model experiments in mice, Ahrén 1655–1665.

Microribonucleic acid‐151‐3p

Circular ribonucleic acid nucleoporin 98 knockdown alleviates high glucose‐induced proliferation, fibrosis, inflammation and oxidative stress in human glomerular mesangial cells by regulating the microribonucleic acid‐151‐3p–high mobility group AT‐hook 2 axis, Dong 1303–1315.

Micro ribonucleicacid‐363

Micro ribonucleic acid‐363 regulates the phosphatidylinositol 3‐kinase/threonine protein kinase axis by targeting NOTCH1 and forkhead box C2, leading to hepatic glucose and lipids metabolism disorder in type 2 diabetes mellitus, Peng 236–248.

Micro RNA

Investigation of umbilical cord serum miRNAs associated with childhood obesity: A pilot study from a birth cohort study, Takatani 1740–1744.

Microvascular complication

Incidence and risk factors of severe non‐proliferative/proliferative diabetic retinopathy: More than a decade follow up in the Tehran Lipids and Glucose Study, Sardarinia 317–327.

Mild obesity

Bariatric surgery versus medical treatment in mildly obese patients with type 2 diabetes mellitus in Japan: Propensity score‐matched analysis on real‐world data, Seki 74–84.

miR‐20b‐5p

CircZNF532 knockdown protects retinal pigment epithelial cells against high glucose‐induced apoptosis and pyroptosis by regulating the miR‐20b‐5p/STAT3 axis, Liang 781–795.

miR‐4,431

miR‐4,431 targets TRIP10/PRKD1 and impairs glucose metabolism, Pan 617–627.

miR‐4,729

Long non‐coding ribonucleic acid ATP2B1‐AS1modulates endothelial permeability through regulating the miR‐4,729–IQGAP2 axis in diabetic retinopathy, Ren 443–452.

Mitochondria

Mitochondrial haplogroups have a better correlation to insulin requirement than nuclear genetic variants for type 2 diabetes mellitus in Taiwanese individuals, Shen 201–208.

Mobile health

High engagement in mobile peer support is associated with better glycemic control in type 1 diabetes: A real‐world study, Liu 1914–1924.

Monogenic diabetes

Precision medicine in diabetes: A non‐invasive prenatal diagnostic test for the determination of fetal glucokinase mutations, Nouspikel 256–261.
Precision diabetes: Lessons learned from maturity‐onset diabetes of the young (MODY), Tosur 465–1471.

Mortality

Effect of diabetes and prediabetes on the development of disability and mortality among middle‐aged Japanese adults: A 22‐year follow up of NIPPON DATA 90, Hoang 1897–1904.

Multi‐metal exposure

Effects of multi‐metal exposure on the risk of diabetes mellitus among people aged 40–75 years in rural areas in southwest China, Zhang 1412–1425.

Multiple daily insulin therapy

Basal insulin requirement in patients with type1 diabetes depends on the age and body mass index, Mitsui 292–298.

Muscle mass

Association between muscle mass, bone mineral density and osteoporosis in type 2 diabetes, Pan 351–358.

Nephropathy

Renal, cardiovascular and safety outcomes of canagliflozin in patients with type 2 diabetes and nephropathy in East and South‐East Asian countries: Results from the Canagliflozin and Renal Events in Diabetes with Established Nephropathy Clinical Evaluation Trial, Wada 54–64.

Nerve conduction

Longitudinal effects of one‐leg standing time on neuropathy outcomes in association with glycemic control in non‐elderly patients with type 2 diabetes, Sugimoto 1039–1051.

Nerve conduction studies

Association between sarcopenia and the severity of diabetic polyneuropathy assessed by nerve conduction studies in Japanese patients with type 2 diabetes mellitus, Mikura 1357–1365.

Neurodevelopment

Neurodevelopmental delay up to the age of 4 years in infants born to women with gestational diabetes mellitus: The Japan Environment and Children's Study, Saito 2054–2062.

Nocturnal hypoglycemia

Comparisons of efficacy and safety in insulin glargine and lixisenatide plus glulisine combination therapy with multiple daily injection therapy in Japanese patients with type 2 diabetes, Kawaguchi 505–514.

NOD mouse

Combination of anti‐CD25 antibody and polyI:C treatment in pregnant NOD mice may be used as ‘pregnancy‐related’ type 1diabetes model, Shimada 1489–1495.

Nonagenarian

Clinical features and sulfonylurea usage among outpatients with diabetes aged ≥90 years in an urban diabetes clinic in Tokyo, Kobori 2010–2017.

Non‐alcoholic fatty liver disease

Non‐alcoholic fatty liver disease and type 2diabetes: An update, Lee 930–940.
Combined evaluation of Fibrosis‐4 index and fatty liver for stratifying the risk for diabetes mellitus, Todo 1577–1584.

Non‐coding RNAs

Non‐coding RNAs underlying the pathophysiological links between type 2 diabetes and pancreatic cancer: A systematic review, Dehghanian 405–428.

Non‐esterified fatty acid

Metabolic state switches between morning and evening in association with circadian clock in people without diabetes, Fujimoto 1496–1505.

Nutrients

Roles of glucose‐dependent insulinotropic polypeptide in diet‐induced obesity, Seino 1122‐‐1128.

Nutritional education

Comparison of the effects of frequent versus conventional nutritional interventions inpatients with type 2 diabetes mellitus: A randomized, controlled trial, Kawabata 271–279.

Obesity

Effects of intermittent very‐low calorie diet on glycemic control and cardiovascular risk factors in obese patients with type 2 diabetes mellitus: A randomized controlled trial, Umphonsathien 156–166.
Metabolic associated fatty liver disease is a risk factor for chronic kidney disease, Hashimoto 308–316.
Pregestational diabetes mediates the association between maternal obesity and the risk of congenital heart defects, Wu 367–374.
A Japanese patient with a 2p25.3 terminal deletion presented with early‐onset obesity, intellectual disability and diabetes mellitus: A case report, Sakaue 391–396.
East Asian perspectives in metabolic and bariatric surgery, Oh 756–761.
Association between maternal gestational diabetes mellitus and high‐sensitivity C‐reactive protein levels in 8‐year‐old children: The Yamanashi Adjunct Study of the Japan Environment and Children's Study(JECS), Sekine 1444–1447.

Obstructive sleep apnea

Therapeutic effect of laparoscopic sleeve gastrectomy on obstructive sleep apnea and relationship of type 2 diabetes in Japanese patients with severe obesity, Yanari 1073–1085.
Obstructive sleep apnea, prediabetes and progression of type 2 diabetes: A systematic review and meta‐analysis, Wang 1396–1411.
Prevalence of obstructive sleep apnea syndrome in hospitalized patients with type 2 diabetes in Beijing, China, Ding 1889–1896.

Offspring

Neurodevelopmental delay up to the age of 4 years in infants born to women with gestational diabetes mellitus: The Japan Environment and Children's Study, Saito 2054–2062.

Onset age

Relationship between onset age of type 2 diabetes mellitus and vascular complications based on propensity score matching analysis, Huang 1062–1072.

Oral antidiabetic drug

Relationship between the early initiation of insulin treatment and diabetic complications inpatients newly diagnosed with type 2 diabetes mellitus in Korea: A nationwide cohort study, Jeon 830–838.

Optical coherence tomography angiography

Measuring the foveal avascular zone in diabetes: A study using optical coherence tomography angiography, Aitchison 668–676.

Organ‐specific insulin sensitivity,

Effects of eicosapentaenoic acid on serum levels of selenoprotein P and organ‐specific insulin sensitivity in humans with dyslipidemia and type 2 diabetes, Takeshita 532–542.

Osteomyelitis

When amputation is not the end of the challenge: A successful therapy for osteomyelitis and soft tissue infection in a patient with type 1 diabetes, Caruso 209–212.

Osteoporosis

Association between muscle mass, bone mineral density and osteoporosis in type 2 diabetes, Pan 351–358.

Oxygen therapy

HbA1c level may be a risk factor for oxygen therapy requirement in patients with coronavirus disease 2019, Yanagisawa 909–917.

Painful diabetic peripheral neuropathy

Prevalence and risk factors for diabetic peripheral neuropathy, neuropathic pain and foot ulceration in the Arabian Gulf region, Ponirakis 1551–1559.

Pancreatectomy

Perioperative C‐peptide index is associated with the status of diabetes management after pancreatectomy, Shikata 1685–1694.

Pancreatic cancer

Non‐coding RNAs underlying the pathophysiological links between type 2 diabetes and pancreatic cancer: A systematic review, Dehghanian 405–428.

Patients

Young‐onset diabetes patients in Thailand: Data from Thai Type 1 Diabetes and Diabetes diagnosed Age before 30 years Registry, Care and Network (T1DDAR CN), Dejkhamron 796–809.

Pediatric diabetes

Precision diabetes: Lessons learned from maturity‐onset diabetes of the young (MODY), Tosur 465–1471.

Peer support

High engagement in mobile peer support is associated with better glycemic control in type 1 diabetes: A real‐world study, Liu 1914–1924.

Percutaneous coronary intervention

Impact of diabetes on coronary physiology evaluated by quantitative flow ratio in patients who underwent percutaneous coronary intervention, Ye 1203–1212.

Perinatal outcome

Trends in maternal characteristics and perinatal outcomes among Japanese pregnant women with type 1 and type 2 diabetes from 1982 to 2020, Fujikawa Shingu 1761–1770.

Peripheral artery disease

The early diagnostic value of ankle‐brachial index combined with feet electrochemical skin conductance for peripheral artery disease in type 2 diabetes, Zhao 525–531.

Peroxisome proliferator‐activated receptor gamma

Ocular expression of cyclin‐dependent kinase5 in patients with proliferative diabetic retinopathy, Sano 628–637.

Pharmacokinetics

Preserved pharmacokinetics and pharmacodynamics of insulin degludec and liraglutide when administered as insulin degludec/liraglutide in a Chinese population, Liu 652–656.

Phenotype

Analysis of clinical phenotypes of neuropathic symptoms in patients with type 2 diabetes: A multicenter study, Kim 1852–1860.

Physical activity

Active commuting, commuting modes and the risk of diabetes: 14‐year follow‐up data from the Hisayama study, Honda 1677–1684.

Physical inactivity

Progressive loss of corneal nerve fibers is associated with physical inactivity and glucose lowering medication associated with weight gain in type 2 diabetes, Ponirakis 1703–1710.

Placenta

Placental expression of glucose transportersGLUT‐1, GLUT‐3, GLUT‐8 and GLUT‐12 in pregnancies complicated by gestational and type 1 diabetes mellitus, Stanirowski 560–570.

Plant sterols

Plant sterol hyperabsorption caused by uncontrolled diabetes in a patient with a heterozygous *ABCG5* variant, Hasebe 1934–1938.

Plasma

Plasma cytokines during pregnancy provide insight into the risk of diabetes in the gestational diabetes risk group, Tagoma 1596–1606.

Polyclonal antiserum

Immunohistochemical detection of enteroviruses in pancreatic tissues of patients with type 1 diabetes using a polyclonal antibody against 2A protease of Coxsackievirus, Jimbo 435–442.

Polyneuropathy

A case of polyneuropathy associated with diabetic ketoacidosis in new‐onset type 1 diabetes, Sada 918–922.

Postprandial hyperglycemia

Low fasting glucose‐to‐estimated average glucose ratio was associated with superior response to insulin degludec/aspart compared with basal insulin in patients with type 2 diabetes, Jang 85–93.

Potassium

Reduction in the magnitude of serum potassium elevation in combination therapy with esaxerenone (CS‐3150) and sodium–glucose cotransporter 2 inhibitor in patients with diabetic kidney disease: Subanalysis of two phase III studies, Shikata 1190–1202.

Precision medicine

Precision medicine in diabetes: A non‐invasive prenatal diagnostic test for the determination of fetal glucokinase mutations, Nouspikel 256–261.

Prediabetes

Association between visceral adiposity index and risk of prediabetes: A meta‐analysis of observational studies, Wang 543–551.
Diet‐induced prediabetes: Effects on the activity of the renin–angiotensin–aldosterone system in selected organs, Mkhize 768–780.
Association of body mass index with risk of prediabetes in Chinese adults: A population‐based cohort study, Chai 1235–1244.
β‐Cell function and body mass index are predictors of exercise response in elderly patients with prediabetes, He 1253–1261.
Obstructive sleep apnea, prediabetes and progression of type 2 diabetes: A systematic review and meta‐analysis, Wang 1396–1411.
Effect of diabetes and prediabetes on the development of disability and mortality among middle‐aged Japanese adults: A 22‐year follow up of NIPPON DATA 90, Hoang 1897–1904.

Pregestational diabetes

Pregestational diabetes mediates the association between maternal obesity and the risk of congenital heart defects, Wu 367–374.

Pregnancy

Association of leukocyte counts in the first trimester with glucose intolerance during pregnancy, Wang 191–200.
Combination of anti‐CD25 antibody and polyI:C treatment in pregnant NOD mice may be used as ‘pregnancy‐related’ type 1diabetes model, Shimada 1489–1495.

Pregnancy outcome

Association of glycated hemoglobin at an early stage of pregnancy with the risk of gestational diabetes mellitus among non‐diabetic women in Japan: The Japan Environment and Children's Study, Sekine 687–695.
Maternal sleep during pregnancy and adverse pregnancy outcomes: A systematic review and meta‐analysis, Wang 1262–1276.

Presarcopenia

Association of subtle alterations in thyroid function with presarcopenia in patients with type 2 diabetes mellitus, Li 696–705.

Prevalence

Prevalence and clinical characteristics of fulminant type 1 diabetes mellitus in Korean adults: A multi‐institutional joint research, Song 47–53.
Prevalence of obstructive sleep apnea syndrome in hospitalized patients with type 2 diabetes in Beijing, China, Ding 1889–1896.

Probiotics

Effects of probiotic *Bifidobacterium bifidum* G9‐1 on the gastrointestinal symptoms of patients with type 2 diabetes mellitus treated with metformin: An open‐label, single‐arm, exploratory research trial, Hata 489–500.

Proliferative diabetic retinopathy

Incidence and risk factors of severe non‐proliferative/proliferative diabetic retinopathy: More than a decade follow up in the Tehran Lipids and Glucose Study, Sardarinia 317–327.

Prospective study

Active commuting, commuting modes and the risk of diabetes: 14‐year follow‐up data from the Hisayama study, Honda 1677–1684.

Protein

Glucose‐dependent insulinotropic polypeptide secretion after oral macronutrient ingestion: The human literature revisited and a systematic study in model experiments in mice, Ahrén 1655–1665.

Public health practice

Abdominal volume index trajectories and risk of diabetes mellitus: Results from the China Health and Nutrition Survey, Hu 868–877.

Pulmonary embolism

Diabetes mellitus and its impact on mortality rate and outcome in pulmonary embolism, Volker H Schmitt 725–737.

Pyroptosis

VX‐765 ameliorates renal injury and fibrosis in diabetes by regulating caspase‐1‐mediated pyroptosis and inflammation, Wen 22–33.

Quantitative flow ratio

Impact of diabetes on coronary physiology evaluated by quantitative flow ratio in patients who underwent percutaneous coronary intervention, Ye 1203–1212.

Quantitative RT‐PCR

Novel method utilizing bisulfite conversion with dual amplification‐refractory mutation system polymerase chain reaction to detect circulating pancreatic β‐cell cfDNA, Okada 1140–1148.

Ramadan

Effect of Ramadan fasting in patients with type 2 diabetes mellitus treated with sodium–glucose cotransporter 2 inhibitors: A systematic review and meta‐analysis, Gad 822–829.

Rapid decliner

Renoprotective effect of additional sodium–glucose cotransporter 2 inhibitor therapy in type 2 diabetes patients with rapid decline and preserved renal function, Sada 1330–1338.

Renal composite outcome

Concomitant treatment with insulin and sodium–glucose cotransporter 2 inhibitors was associated with the renal composite outcome in Japanese patients with type 2 diabetes and chronic kidney disease: A propensity score‐matched analysis, Toyoda 1520–1527.

Renin–angiotensin–aldosterone system

Diet‐induced prediabetes: Effects on the activity of the renin–angiotensin–aldosterone system in selected organs, Mkhize 768–780.

Retrospective study

Impact of diabetes and Krebs von den Lungen‐6 on coronavirus disease 2019 severity: A single‐center study from Japan, Yakushiji 1277–1285.

Reverse clinical inertia

Clinical features and sulfonylurea usage among outpatients with diabetes aged ≥90 years in an urban diabetes clinic in Tokyo, Kobori 2010–2017.

Risk factor

HbA1c level may be a risk factor for oxygen therapy requirement in patients with coronavirus disease 2019, Yanagisawa 909–917.

Risk factors

Acute kidney injury in Japanese type 2 diabetes patients receiving sodium–glucose cotransporter 2 inhibitors: A nationwide cohort study, Horii 42–46.
HbA1c, blood pressure, and cholesterol control in adults with diabetes: A report card for Kuwait, Alkandari 1732–1739.

Risk model

Non‐laboratory‐based risk assessment model for case detection of diabetes mellitus and pre‐diabetes in primary care, Dong 1374–1386.

Sarcopenia

Association between sarcopenia and the severity of diabetic polyneuropathy assessed by nerve conduction studies in Japanese patients with type 2 diabetes mellitus, Mikura 1357–1365.
Sarcopenia is associated with the Geriatric Nutritional Risk Index in elderly patients with poorly controlled type 2 diabetes mellitus, Matsuura 1366–1373.
Poor glycemic control rather than types of diabetes is a risk factor for sarcopenia in diabetes mellitus: The MUSCLES‐DM study, Hiromine 1881–1888.

Scanning

Frequent scanning using flash glucose monitoring contributes to better glycemic control in children and adolescents with type 1 diabetes, Urakami 185–190.

Selenoprotein P

Effects of eicosapentaenoic acid on serum levels of selenoprotein P and organ‐specific insulin sensitivity in humans with dyslipidemia and type 2 diabetes, Takeshita 532–542.

Serotonin

Serotonin in the regulation of systemic energy metabolism, Moon 1639–1645.

Severe acute respiratory syndrome coronavirus 2

New‐onset fulminant type 1 diabetes after severe acute respiratory syndrome coronavirus 2 vaccination: A case report, Sasaki 1286–1289.

Severe hypoglycemia

Trends of severe hypoglycemia in patients with type 2 diabetes in Korea: A longitudinal nationwide cohort study, Yun 1438–1443.
Adherence to healthy lifestyle behaviors as a preventable risk factor for severe hypoglycemia in people with type 2 diabetes: A longitudinal nationwide cohort study, Yun 1533–1542.
Factors associated with hypoglycemia unawareness and severe hypoglycemia in type 1 diabetes mellitus patients, Takagi 2018–2026.

Severe progression

Glycated hemoglobin level on admission associated with progression to severe disease in hospitalized patients with non‐severe coronavirus disease 2019, Numaguchi 1779–1787.

Sex

Metabolic Score for Insulin Resistance, a novel score to evaluate insulin sensitivity, is associated with the urinary albumin‐to‐creatinine ratio in Chinese adults: A cross‐sectional REACTION study, Su 1222–1234.

Sexual dysfunction

Gaps in the management of diabetes in Asia: A need for improved awareness and strategies in men's sexual health, Kang 1945–1957.

Shear wave elastography

Quantitative evaluation of insulin‐induced abdominal subcutaneous dystrophic tissue using shear wave elastography, Sato 1004–1010.

Sitagliptin

Efficacy and safety of sitagliptin and insulin for latent autoimmune diabetes in adults: A systematic review and meta‐analysis, Lin 1506–1519.

Skin thickness

Repeated insulin injection without site rotation affects skin thickness – ultrasonographic and histological evaluation, Murao 997–1003.

Sleep

Maternal sleep during pregnancy and adverse pregnancy outcomes: A systematic review and meta‐analysis, Wang 1262–1276.

Slope of estimated glomerular filtration rate decline

Renoprotective effect of additional sodium–glucose cotransporter 2 inhibitor therapy in type 2 diabetes patients with rapid decline and preserved renal function, Sada 1330–1338.

Small dense low‐density lipoprotein cholesterol

Dyslipidemia in diabetic kidney disease classified by proteinuria and renal dysfunction: A cross‐sectional study from a regional diabetes cohort, Hirano 657–667.

Social restrictions

Year‐long effects of COVID‐19 restrictions on glycemic control and body composition inpatients with glucose intolerance in Japan: A single‐center retrospective study, Tsukaguchi 2063–2072.

Sodium–glucose cotransporter 2 inhibitor

Effect of Ramadan fasting in patients with type 2 diabetes mellitus treated with sodium–glucose cotransporter 2 inhibitors: A systematic review and meta‐analysis, Gad 822–829.
Adenosine/adenosine type 1 receptor signaling pathway did not play dominant roles on the influence of sodium–glucose cotransporter 2 inhibitor in the kidney of bovine serum albumin‐overloaded streptozotocin‐induced diabetic mice, Shimada 955–964.
Current status of diabetes treatment in Miyazaki Prefecture, Japan: Results of a questionnaire survey conducted in 2016 and 2020, Uehira 1011–1020.
Reduction in the magnitude of serum potassium elevation in combination therapy with esaxerenone (CS‐3150) and sodium–glucose cotransporter 2 inhibitor in patients with diabetic kidney disease: Subanalysis of two phase III studies, Shikata 1190–1202.
Renoprotective effect of additional sodium–glucose cotransporter 2 inhibitor therapy in type 2 diabetes patients with rapid decline and preserved renal function, Sada 1330–1338.
Effect of sodium–glucose cotransporter 2 inhibitor medication on new prescriptions of antihypertensives, antigout/antihyperuricemics and antidyslipidemics in Japan: Analysis using the JMDC Claims Database, Gouda 1842–1851.
Effect of canagliflozin on white blood cell counts in patients with type 2 diabetes and heart failure: A subanalysis of the randomized CANDLE trial, Tanaka 1990–1999.
Real‐world risk of lower‐limb amputation associated with sodium–glucose cotransporter 2 inhibitors versus metformin: A propensity score‐matched model analysis in Japan, Mizutani 2000–2009.

Sodium–glucose cotransporter 2 inhibitors

Acute kidney injury in Japanese type 2 diabetes patients receiving sodium–glucose cotransporter 2 inhibitors: A nationwide cohort study, Horii 42–46.
Sodium–glucose cotransporter 2 inhibitors and anemia among diabetes patients in real clinical practice, Murashima 638–646.
Healthcare resource utilization in patients treated with empagliflozin in East Asia, Sheu 810–821.
Sodium–glucose cotransporter 2 inhibitors do not increase the risk of fractures in real‐world clinical practice in Korea: A national observational cohort study, Ha 986–996.
Efficacy and safety of once‐weekly semaglutide in Japanese individuals with type 2 diabetes by baseline age and body mass index, Yabe 1161–1174.
Concomitant treatment with insulin and sodium–glucose cotransporter 2 inhibitors was associated with the renal composite outcome in Japanese patients with type 2 diabetes and chronic kidney disease: A propensity score‐matched analysis, Toyoda 1520–1527.

Soluble tumor necrosis factor‐α receptor 1

Synergistic association of the copper/zinc ratio under inflammatory conditions with diabetic kidney disease in patients with type 2 diabetes: The Asahi Diabetes Complications Study, Takao 299–307.

STAT3

CircZNF532 knockdown protects retinal pigment epithelial cells against high glucose‐induced apoptosis and pyroptosis by regulating the miR‐20b‐5p/STAT3 axis, Liang 781–795.

Steroid

Factors affecting glycemic control in diabetes mellitus complicated by autoimmune pancreatitis, Harai 1387–1395.

Stigma

A survey of clinical physician's perceptions of stigma and advocacy in patients with type 2diabetes in Kanagawa, Japan, Matsuzawa 2073–2080.
Stigma evaluation for diabetes and other chronic non‐communicable disease patients: Development, validation and clinical use of stigma scale–The Kanden Institute Stigma Scale, Tanaka 2081–2090.

Subcutaneous insulin resistance syndrome

Case of subcutaneous insulin resistance syndrome treated with ultra‐rapid insulin lispro, Ishii 588–591.

Subtypes

Relation between the insulin lowering rate and changes in bone mineral density: Analysis among subtypes of type 1 diabetes mellitus, Suzuki 1585–1595.

Sulfonylurea

Clinical features and sulfonylurea usage among outpatients with diabetes aged ≥90 years in an urban diabetes clinic in Tokyo, Kobori 2010–2017.

Targets

HbA1c, blood pressure, and cholesterol control in adults with diabetes: A report card for Kuwait, Alkandari 1732–1739.

Telemedicine

Diabetes management by either telemedicine or clinic visit improved glycemic control during the coronavirus disease 2019 pandemic state of emergency in Japan, Onishi 386–390.
Substitution of telemedicine for clinic visit during the COVID‐19 pandemic of 2020: Comparison of telemedicine and clinic visit, Onishi 1617–1625.

Thai Type 1 Diabetes and Diabetes diagnosed Age before 30 years Registry, Care and Network

Young‐onset diabetes patients in Thailand: Data from Thai Type 1 Diabetes and Diabetes diagnosed Age before 30 years Registry, Care and Network (T1DDAR CN), Dejkhamron 796–809.

Thyroid function

Association of subtle alterations in thyroid function with presarcopenia in patients with type 2 diabetes mellitus, Li 696–705.

Time in range

Frequent scanning using flash glucose monitoring contributes to better glycemic control in children and adolescents with type 1 diabetes, Urakami 185–190.
Comparison of glucose time in range and area under curve in range in relation to risk of diabetic retinopathy in type 2 diabetes patients, Wang 1543–1550.
Time in range assessed by capillary blood glucose in relation to insulin sensitivity and β‐cell function in patients with type 2 diabetes mellitus: A cross‐sectional study in China, Ye 1825–1833.

Tocilizumab

Development of type 1 diabetes in a patient treated with anti‐interleukin‐6 receptor antibody for rheumatoid arthritis, Kawasaki 738–740.

Total internal reflection fluorescence microscope

Direct visualization of glucagon‐like peptide‐1secretion by fluorescent fusion proteins, Tsuzuki 1134–1139.

Transient receptor potential melastatin 2

A novel mechanism of imeglimin‐mediated insulin secretion via the cADPR‐TRP channel pathway, Funazaki 34–41.

Treatment

Efficacy and safety of once‐weekly semaglutide in Japanese individuals with type 2 diabetes in the SUSTAIN 1, 2, 5 and 9 trials: Post‐hoc analysis, Araki 1971–1980.

Treatment‐transplantation

β‐Cell replacement therapy for the cure of diabetes, Lee 1798–1802.

Triglyceridemia

Oral fat tolerance testing identifies abnormal pancreatic β‐cell function and insulin resistance in individuals with normal glucose tolerance, Liu 1805–1813.

Type 1 and type 2 diabetes

Poor glycemic control rather than types of diabetes is a risk factor for sarcopenia in diabetes mellitus: The MUSCLES‐DM study, Hiromine 1881–1888.

Type 1 diabetes

Basal insulin requirement in patients with type1 diabetes depends on the age and body mass index, Mitsui 292–298.
Use of automated insulin delivery systems in people with type 1 diabetes fasting during Ramadan: An observational study, Aldibbiat 647–651.
Usefulness of subclassification of adult diabetes mellitus among inpatients in Japan, Saito 706–713.
Development of type 1 diabetes in a patient treated with anti‐interleukin‐6 receptor antibody for rheumatoid arthritis, Kawasaki 738–740.
A case of polyneuropathy associated with diabetic ketoacidosis in new‐onset type 1 diabetes, Sada 918–922.
Newly developed type 1 diabetes after coronavirus disease 2019 vaccination: A case report, Sasaki 1105–1108.
Novel method utilizing bisulfite conversion with dual amplification‐refractory mutation system polymerase chain reaction to detect circulating pancreatic β‐cell cfDNA, Okada 1140–1148.
Type 1 diabetes mellitus following COVID‐19 RNA‐based vaccine, Sakurai 1290–1292.
Point‐of‐care sural nerve conduction could predict the presence of cardiovascular autonomic neuropathy in type 1 diabetes mellitus, Nattero‐Chávez 1347–1356.
Combination of anti‐CD25 antibody and polyI:C treatment in pregnant NOD mice may be used as ‘pregnancy‐related’ type 1diabetes model, Shimada 1489–1495.
High prevalence of depressive symptoms among people with pediatric‐onset and adolescent‐onset type 1 diabetes: A cross‐sectional analysis of the Diabetes Study from the Center of Tokyo Women's Medical University, Takaike 1626–1632.
Comparison of the clinical effects of intermittently scanned and real‐time continuous glucose monitoring in children and adolescents with type 1 diabetes: A retrospective cohort study, \ Urakami 1745–1752.
β‐Cell replacement therapy for the cure of diabetes, Lee 1798–1802.
High engagement in mobile peer support is associated with better glycemic control in type 1 diabetes: A real‐world study, Liu 1914–1924.
Plant sterol hyperabsorption caused by uncontrolled diabetes in a patient with a heterozygous ABCG5 variant, Hasebe 1934–1938.
Factors associated with hypoglycemia unawareness and severe hypoglycemia in type 1 diabetes mellitus patients, Takagi 2018–2026.

Type 1 diabetes mellitus

Impact of flash glucose monitoring on glycemic control varies with the age and residual β‐cell function of patients with type 1 diabetes mellitus, Zhang 552–559.
Comparison of continuous subcutaneous insulin infusion treatment and multiple daily injection treatment on the progression of diabetic complications in Japanese patients with juvenile‐onset type 1 diabetes mellitus, Masuda 1528–1532.
Relation between the insulin lowering rate and changes in bone mineral density: Analysis among subtypes of type 1 diabetes mellitus, Suzuki 1585–1595.
Trends in maternal characteristics and perinatal outcomes among Japanese pregnant women with type 1 and type 2 diabetes from 1982 to 2020, Fujikawa Shingu 1761–1770.

Type 2 diabetes

Diversity of pathophysiology in type 2 diabetes shown by islet pathology, Mizukami 6–13.
Micro ribonucleic acid‐363 regulates the phosphatidylinositol 3‐kinase/threonine protein kinase axis by targeting NOTCH1 and forkhead box C2, leading to hepatic glucose and lipids metabolism disorder in type 2 diabetes mellitus, Peng 236–248.
Efficacy of acceptance and commitment therapy for people with type 2 diabetes: Systematic review and meta‐analysis, Sakamoto 262–270.
Retrospective nationwide study on the trends in first‐line antidiabetic medication for patients with type 2 diabetes in Japan, Bouchi 280–291.
Non‐coding RNAs underlying the pathophysiological links between type 2 diabetes and pancreatic cancer: A systematic review, Dehghanian 405–428.
Fasting plasma glucose level in the range of 90–99 mg/dL and the risk of the onset of type 2 diabetes: Population‐based Panasonic cohort study 2, Munekawa 453–459.
Dipeptidyl peptidase‐4 inhibitor and insulin combination treatment in type 2 diabetes and chronic kidney disease: A meta‐analysis, Zhou 468–477.
Factors associated with the degree of glycemic deterioration among patients with type 2 diabetes who dropped out of diabetes care: A longitudinal analysis using medical claims and health checkup data in Japan, Ihana‐Sugiyama 571–579.
Validity and reliability of the Japanese version of the diabetes knowledge test among in‐patients with type 2 diabetes, Minami 580–587.
Association of subtle alterations in thyroid function with presarcopenia in patients with type 2 diabetes mellitus, Li 696–705.
Usefulness of subclassification of adult diabetes mellitus among inpatients in Japan, Saito 706–713.
Non‐alcoholic fatty liver disease and type 2diabetes: An update, Lee 930–940.
Efficacy and safety of oral semaglutide in Japanese patients with type 2 diabetes: A subgroup analysis by baseline variables in the PIONEER 9 and PIONEER 10 trials, Yabe 975–985.
Visit‐to‐visit variability in albuminuria predicts renal function deterioration in patients with type 2 diabetes, Lin 1021–1029.
Relationship of coffee consumption with a decline in kidney function among patients with type 2 diabetes: The Fukuoka Diabetes Registry, Komorita 1030–1038.
Therapeutic effect of laparoscopic sleeve gastrectomy on obstructive sleep apnea and relationship of type 2 diabetes in Japanese patients with severe obesity, Yanari 1073–1085.
Effect of glucagon‐like peptide‐1 receptor agonists on fat distribution in patients with type 2 diabetes: A systematic review and meta‐analysis, Duan 1149–1160.
Association between vitamin D status and diabetic foot in patients with type 2 diabetes mellitus, Tang 1213–1221.
Association between changes in bodyweight and cardiovascular disease risk factors among obese Japanese patients with type 2 diabetes, Yoshimura 1560–1566.
Abdominal aortic calcification is associated with Fibrosis‐4 index and low body mass index in type 2 diabetes patients: A retrospective cross‐sectional study, Togashi 1861–1872.
Prevalence of obstructive sleep apnea syndrome in hospitalized patients with type 2 diabetes in Beijing, China, Ding 1889–1896.
Real‐world risk of lower‐limb amputation associated with sodium–glucose cotransporter 2 inhibitors versus metformin: A propensity score‐matched model analysis in Japan, Mizutani 2000–2009.

Type 2 diabetes mellitus

Bariatric surgery versus medical treatment in mildly obese patients with type 2 diabetes mellitus in Japan: Propensity score‐matched analysis on real‐world data, Seki 74–84.
Effectiveness and safety of ertugliflozin for type 2 diabetes: A meta‐analysis of data from randomized controlled trials, Zhang 478–488.
Case of subcutaneous insulin resistance syndrome treated with ultra‐rapid insulin lispro, Ishii 588–591.
miR‐4,431 targets TRIP10/PRKD1 and impairs glucose metabolism, Pan 617–627.
Preserved pharmacokinetics and pharmacodynamics of insulin degludec and liraglutide when administered as insulin degludec/liraglutide in a Chinese population, Liu 652–656.
East Asian perspectives in metabolic and bariatric surgery, Oh 756–761.
Effect of Ramadan fasting in patients with type 2 diabetes mellitus treated with sodium–glucose cotransporter 2 inhibitors: A systematic review and meta‐analysis, Gad 822–829.
Predictive ability of current machine learning algorithms for type 2 diabetes mellitus: A meta‐analysis, Kodama 900–908.
Sodium–glucose cotransporter 2 inhibitors do not increase the risk of fractures in real‐world clinical practice in Korea: A national observational cohort study, Ha 986–996.
Relationship between onset age of type 2 diabetes mellitus and vascular complications based on propensity score matching analysis, Huang 1062–1072.
A randomized trial to investigate the efficacy and safety of once‐daily liraglutide 1.8 mg in Japanese adults with type 2 diabetes exhibiting an inadequate response to liraglutide 0.9 mg, Seino 1321–1329.
Sarcopenia is associated with the Geriatric Nutritional Risk Index in elderly patients with poorly controlled type 2 diabetes mellitus, Matsuura 1366–1373.
Transition of cardiometabolic status and the risk of type 2 diabetes mellitus among middle‐aged and older Chinese: A national cohort study, Qiu 1426–1437.
Current status of low‐density lipoprotein cholesterol for primary prevention of coronary artery disease in late‐stage elderly persons with type 2 diabetes mellitus: A retrospective, single‐center study, Yamamoto 1567–1576.
Association of ideal cardiovascular health metrics and incident type 2 diabetes mellitus among an urban population of Iran: One decade follow up in the Tehran Lipid and Glucose Study, Asgari 1711–1722.
C1q tumor necrosis factor‐related protein 4 is associated with coronary artery disease in patients with type 2 diabetes, Gao 1723–1731.
Trends in maternal characteristics and perinatal outcomes among Japanese pregnant women with type 1 and type 2 diabetes from 1982 to 2020, Fujikawa Shingu 1761–1770.
Clinical course of adolescents with type 2 diabetes mellitus: A nationwide cohort study in Taiwan, Yen 1905–1913.
Effect of canagliflozin on the decline of estimated glomerular filtration rate in chronic kidney disease patients with type 2 diabetes mellitus: A multicenter, randomized, double‐blind, placebo‐controlled, parallel‐group, phase III study in Japan, Wada 1981–1989.
Impact of healthy lifestyle on the risk of type 2 diabetes mellitus in southwest China: A prospective cohort study, Wu 2091–210.

Ultra‐rapid insulin lispro

Case of subcutaneous insulin resistance syndrome treated with ultra‐rapid insulin lispro, Ishii 588–591.

Ultrasound

Quantitative evaluation of insulin‐induced abdominal subcutaneous dystrophic tissue using shear wave elastography, Sato 1004–1010.

Vaccination

New‐onset fulminant type 1 diabetes after severe acute respiratory syndrome coronavirus 2 vaccination: A case report, Sasaki 1286–1289.

Validation

Appropriate definition of diabetes using an administrative database: A cross‐sectional cohort validation study, Nishioka 249–255.

Vaccine

Newly developed type 1 diabetes after coronavirus disease 2019 vaccination: A case report, Sasaki 1105–1108.

Venous thromboembolism

Diabetes mellitus and its impact on mortality rate and outcome in pulmonary embolism, Volker H Schmitt 725–737.

Visceral adipose index

Association between visceral adiposity index and risk of prediabetes: A meta‐analysis of observational studies, Wang 543–551.

Vision impairment

Impact of vision and hearing impairments on risk of cardiovascular outcomes and mortality in patients with type 2 diabetes: A nationwide cohort study, Jung 515–524.

Visit‐to‐visit variability

Visit‐to‐visit variability in albuminuria predicts renal function deterioration in patients with type 2 diabetes, Lin 1021–1029.

Visual impairment

Incidence and risk factors of severe non‐proliferative/proliferative diabetic retinopathy: More than a decade follow up in the Tehran Lipids and Glucose Study, Sardarinia 317–327.

Vitamin D deficiency

Association between vitamin D status and diabetic foot in patients with type 2 diabetes mellitus, Tang 1213–1221.

VX‐765

VX‐765 ameliorates renal injury and fibrosis in diabetes by regulating caspase‐1‐mediated pyroptosis and inflammation, Wen 22–33.

Weighted gene co‐expression network analysis

Application of weighted gene co‐expression network analysis to identify novel key genes in diabetic nephropathy, Wang 112–124.

Werner syndrome

Werner syndrome presenting as early‐onset diabetes: A case report, Wang 592–598.

White blood cell

Effect of canagliflozin on white blood cell counts in patients with type 2 diabetes and heart failure: A subanalysis of the randomized CANDLE trial, Tanaka 1990–1999.

Whole grain

Effects of whole grain intake on glycemic control: A meta‐analysis of randomized controlled trials, Li 1814–1824.

*WRN* gene

Werner syndrome presenting as early‐onset diabetes: A case report, Wang 592–598.

Young adult

Reimagining care for young adults living with type 1 diabetes, Morrissey 1294–1299.

Young‐onset diabetes

Young‐onset diabetes patients in Thailand: Data from Thai Type 1 Diabetes and Diabetes diagnosed Age before 30 years Registry, Care and Network (T1DDAR CN), Dejkhamron 796–809.

